# TREM2 acts as a tumor suppressor in hepatocellular carcinoma by targeting the PI3K/Akt/β-catenin pathway

**DOI:** 10.1038/s41389-018-0115-x

**Published:** 2019-01-25

**Authors:** Wenqing Tang, Bei Lv, Biwei Yang, Yukai Chen, Feifei Yuan, Lijie Ma, She Chen, Si Zhang, Jinglin Xia

**Affiliations:** 1 Liver Cancer Institute, Zhongshan Hospital, Fudan University, Shanghai, China; 2 Liver Surgery Department, Liver Cancer Institute, Zhongshan Hospital, Key Laboratory of Carcinogenesis and Cancer Invasion, Ministry of Education, Fudan University, Shanghai, China; 3 Key Laboratory of Glycoconjugate Research Ministry of Public Health, Department of Biochemistry and Molecular Biology, Shanghai Medical College, Fudan University, Shanghai, China; 4 Minhang Hospital, Fudan University, Shanghai, China

## Abstract

Triggering receptor expressed on myeloid cells 2 (TREM2) is involved in nonmalignant pathological processes. However, TREM2’s function in malignant diseases, especially in hepatocellular carcinoma (HCC) remains unknown. In the present study, we report that TREM2 is a novel tumor suppressor in HCC. TREM2 expression was obviously decreased in hepatoma cells (especially metastatic HCC cells), and in most human HCC tissues (especially extrahepatic metastatic tumors). Reduced tumor TREM2 expression was correlated with poor prognosis of HCC patients, and with aggressive pathological features (BCLC stage, tumor size, tumor encapsulation, vascular invasion, and tumor differentiation). *TREM2* knockdown substantially promoted cell growth, migration, and invasion in vitro and in vivo, while *TREM2* overexpression produced the opposite effect. TREM2 suppressed HCC metastasis by inhibiting epithelial-mesenchymal transition, accompanied by abnormal expression of epithelial and mesenchymal markers. Further study revealed that downregulation of TREM2 in HCC was regulated by miR-31-5p. Moreover, by directly interacting with β-catenin, TREM2 attenuated oncogenic and metastatic behaviors by inhibiting Akt and GSK3β phosphorylation, and activating β-catenin. TREM2 suppressed carcinogenesis and metastasis in HCC by targeting the PI3K/Akt/β-catenin pathway. Thus, we propose that TREM2 may be a candidate prognostic biomarker in malignant diseases and TREM2 restoration might be a prospective strategy for HCC therapy.

## Introduction

As one of the most common cancers, hepatocellular carcinoma (HCC) is the third leading cause of death from cancer worldwide^1^. Although the survival of HCC patients has improved because of advances in surgical techniques and locoregional therapies, long-term survival rates after surgical resection remain low. Metastasis is the main reason for the high mortality of patients with HCC after surgical resection^2^. Therefore, it is imperative to explore the underlying molecular mechanisms of HCC metastasis.

Epithelial-mesenchymal transition (EMT), a process in which epithelial cells transdifferentiate into motile mesenchymal cells, pathologically leads to fibrosis and cancer progression. The multi-stage process of EMT consists of the gradual remodeling of epithelial cell architecture and functional capabilities. Cells lose the apical-basal cell polarity and epithelial cell–cell junctions, and transform to a low proliferation state with a spindle-like cell shape and with enhanced capacity of cell migration, invasion, and survival^3^. This switch in cell differentiation and behavior is mediated by several critical transcription factors, like snail, slug, and twist, of which the functions are finely regulated at the transcriptional, translational, and posttranslational levels. The reprogramming of gene expression during EMT, along with non-transcriptional changes, are triggered and regulated by signaling pathways that respond to extracellular cues^4^.

Triggering receptor expressed on myeloid cells (TREM) transmembrane proteins, a novel pattern recognition receptor family, play vital roles in regulating inflammation and immune response through their association with adaptor proteins^5^. To date, in humans, TREM1 and TREM2 have been the most widely studied; they share a similar structure and both couple to the transmembrane adaptor molecule, DNAX-activation protein 12 (DAP12) via electrostatic interaction to transduce signals^6,7^. TREM1 is commonly considered to be an enhancer of immune responses, but TREM2 is considered to be a protective negative regulator of inflammation^8,9^. TREM2 is predominantly found on macrophages, microglia, osteoclasts, and dendritic cells^10^. The *TREM2* gene located on human chromosome 6p21.1 encodes a 230 amino acid protein consists of an extracellular immunoglobulin-like domain, a transmembrane domain, and a cytoplasmic tail^11^. TREM2-mediated signaling occurs through phosphorylation of tyrosine residues within the immunoreceptor tyrosine-based activation motif in cytoplasmic domain of DAP12 via Src kinases^12^. This in turn recruits spleen associated tyrosine kinase (SYK) via Src homology domain 2 and subsequently activates the downstream target genes. TREM2 ligands are not completely known, although recently, it was reported that TREM2 binds to microbial products like lipopolysaccharide, gram-negative and gram-positive bacteria^13^, and apolipoprotein E^14^.

To date, most studies on TREM2 have focused on its role in inflammation. TREM2 suppressed Toll-like receptor (TLR) signaling mediated by the adaptor protein myeloid differentiation primary-response gene 88 (MYD88) in mouse macrophages, thus attenuating the inflammatory response^9,15^. TREM2-deficient macrophages displayed impaired induction of the pro-inflammatory cytokines interleukin-6 (IL-6) and tumor necrosis factor alpha (TNF-α) after treatment with the TLR ligands^9^. TREM2-deficient monocyte-derived dendritic cells showed enhanced TLR-mediated maturation and antigen-specific T-cell proliferation^16^. Moreover, TREM2 regulated the mucosal inflammatory response^17^. Microglial cells which lack the DAP12-associated TREM-2 receptor released higher amounts of inflammatory cytokines TNFα and nitric oxide synthase 2 (NOS2)^18^. In addition, TREM2-deficient dendritic cells showed a decreased capacity of generating pro-inflammatory cytokines (IL-6, IL-10, and TNF) in response to bacteria-associated antigens^16^. TREM2 deficiency attenuated neuroinflammation and protects against neurodegeneration^19^. Besides playing a critical role in immune responses, TREM2 was involved in a variety of other biological processes, including osteoclastogenesis^20^, brain homeostasis^21^, and phagocytosis^22^.

The function and mechanism of TREM2 in nonmalignant pathological conditions has been broadly studied. However, up to now, there are few reports on the role of TREM2 in malignant diseases, especially in HCC. In this study, we clarified the significance of TREM2 in HCC metastasis via clinical specimens, in vitro assays, and mice models. We discovered that downregulation of *TREM2* expression in human HCC tissues was correlated with accelerated metastasis and worse survival. Overexpression of *TREM2* in hepatoma cells remarkably prevented tumor metastasis in vitro and in vivo. A further mechanistic study showed TREM2 could interact with β-catenin directly. Moreover, we present evidence that TREM2 prevented HCC metastasis by directly targeting the phosphatidylinositol-4,5-bisphosphate 3-kinase (PI3K)/AKT serine/threonine kinase (Akt)/β-catenin pathway.

## Results

### Decreased TREM2 expression in HCC cells and human HCC

**Fig. 1:**
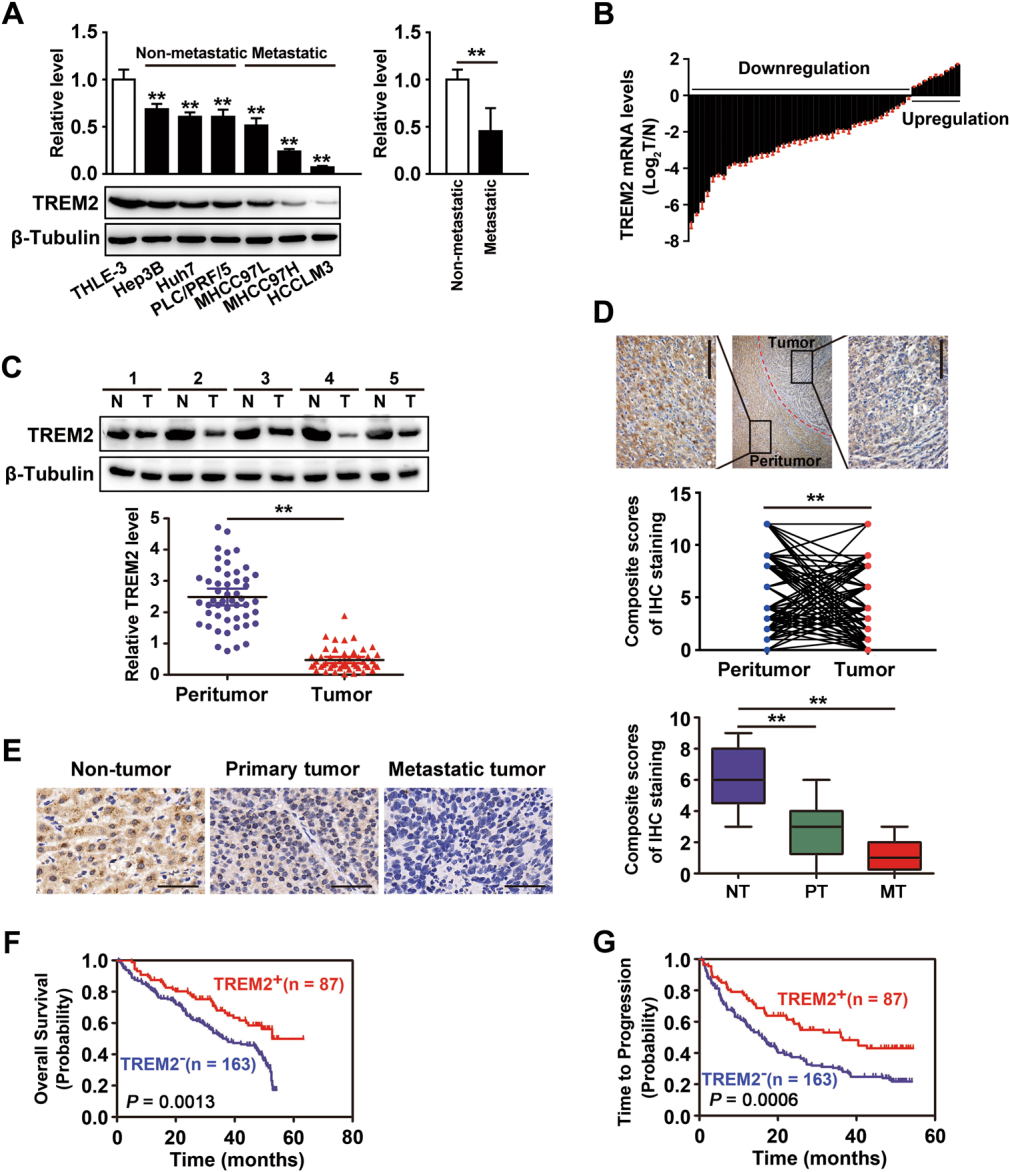
The expression pattern of TREM2 in HCC cell lines and tissues, and its correlation with HCC prognosis. **a** *TREM2* expression in THLE-3 and six hepatoma cells. Left panel: Compared with that in the normal liver cell line THLE-3, TREM2 expression was obviously decreased in hepatoma cells. Right panel: Compared with that in hepatoma cells with low metastatic potential, TREM2 expression was obviously decreased in hepatoma cells with high metastatic potential. Quantification of western blotting data was from three separate experiments. **b** mRNA levels of *TREM2* in 50 cases of paired liver tissue samples. Data were from a representative experiment carried out in triplicate. **c** Protein levels of *TREM2* in 50 cases of clinical samples. Quantification of western blotting data was from three separate experiments. **d** TREM2 staining in tumor tissues and paired adjacent non-tumor liver tissues. Upper panel: Representative IHC staining pattern of TREM2. Lower panel: Scores of IHC staining of TREM2 in 250 samples. Scale bar, 50 μm. **e** Progressive reduce of TREM2 expression level in 40 cases of non-tumor liver tissue, primary HCC, and venous metastasis of the same case. Left panel: Typical patterns of TREM2 staining. Scale bar, 50 μm. NT, adjacent non-tumorous tissues; PT, primary tumor tissues; MT, metastatic tumor tissues. Right panel: Scores of immunochemistry staining of *TREM2* expression. **f** Kaplan–Meier curve of overall survival (OS) for *TREM2* expression. **g** Kaplan–Meier curve of time to progression (TTP) for *TREM2* expression. **P *<* 0.01

We explored TREM2 expression in six HCC cell lines and an immortalized human hepatocyte liver cells (THLE-3). Compared with THLE-3, TREM2 protein levels were markedly reduced in HCC cell lines (Hep3B, Huh7, and PLC/PRF/5) with low metastatic and especially lower in the three HCC cell lines (MHCC97L, MHCC97H, and HCCLM3) with high metastatic potential (Fig. 1a). To investigate TREM2 expression profile in human HCC, quantitative polymerase chain reaction (qPCR) and western blotting was conducted in 50 paired non-tumorous and tumor tissues. The result showed that TREM2 level was obviously downregulated in tumor tissues compared with that in the non-tumor tissues at mRNA and protein levels (Fig. 1b, c). Moreover, immunohistochemistry was performed in 250 paired non-tumor and tumor tissues and 40 cases of samples comprising non-tumor livers, primary HCCs, and venous metastasis from the same patients. Weak TREM2 staining was seen in the tumor tissues, with strong TREM2 staining observed in the non-tumor tissues. The semi-quantitative scores analysis of immunohistochemical (IHC) staining implied that TREM2 was markedly downregulated in tumor compared with that in matched non-tumor tissues (Fig. 1d). In addition, the analysis of TREM2 staining verified that TREM2 expression gradually decreased in the order non-tumor livers, primary tumors, and metastatic tumors (Fig. 1e).

### Loss of TREM2 expression is correlated with poor prognosis of HCC

A cohort of subjects comprising 250 patients with HCC was studied, among which 160 patients were confirmed to have tumor recurrence, and 139 had died by the end of follow-up. The clinical characteristics of the patients were listed in Supplementary Table 1. The tumor TREM2 levels upon IHC were evaluated using the composite expression score (CES) described in the methods. High and low expression of TREM2 was estimated by receiver operating characteristic (ROC) curve analysis, which indicated that the optimal cutoff value of CES was 4 (Supplementary Fig. 1). Therefore, a CES > 4 indicated positive and high expression of TREM2, while CES ≤ 4 indicated negative and low expression. Clinicopathological analysis revealed that TREM2 downregulation was significantly correlated with aggressive pathological features, like BCLC stage, tumor size, tumor encapsulation, vascular invasion and tumor differentiation (Supplementary Table 2). The data from Kaplan–Meier curves showed that HCC patients with negative TREM2 expression in their tumor tissues had shorter survival and increased recurrence than those with positive expression. TREM2 expression in tumor was significantly associated with overall survival (OS) (*P* *=* 0.0013) and time to progression (TTP) (*P* = 0.0006, respectively) (Fig. 1f, g). Univariate analysis implied that reduced TREM2 protein levels were significantly correlated with OS (*P* = 0.001) and TTP (*P* = 0.001). Other factors that affected on OS were Barcelona Clinic Liver Cancer (BCLC) stage, tumor encapsulation, tumor size, and tumor multiplicity. Other factors that were correlated with TTP included BCLC stage, tumor encapsulation, tumor size, tumor multiplicity, and vascular invasion (Supplementary Table 3). Moreover, multivariate analysis showed that TREM2 expression correlated significantly with OS (*P* = 0.024) and TTP (*P* = 0.018); and patients who have negative TREM2 expression were more prone to die and relapse than TREM2-positive ones (Table 1). To validate the prognostic value of a low TREM2 level in HCC, we conducted survival analysis in a different dataset comprising of 135 patients with HCC, of which 79 had tumors with low TREM2 expression and 56 had tumors with high TREM2 expression. The clinicopathologic features of the patients were listed in Supplementary Table 4. The results indicated that low TREM2 level was correlated with poor prognosis of HCC (*P* < 0.0001 for OS and *P* = 0.0266 for TTP) (Supplementary Fig. 2), which was in accordance with our result of the first cohort. These observations implied a tumor suppressor role of TREM2 in HCC development.

**Table 1:** Multivariate analyses of factors correlated with overall survival (OS) and time to progression (TTR)

	HR (95% CI)	*P* values
OS		
BCLC stage (B/C vs. A)	0.931 (0.555–1.562)	0.786
Tumor encapsulation (complete vs. none)	0.720 (0.511–1.014)	0.060
Tumor size, cm (>5 vs. ≤5)	2.020 (1.213–3.365)	**0.007**
Tumor multiplicity (multiple vs. single)	1.653 (1.102–2.480)	**0.015**
TREM2 tumor (positive vs. negative)	0.631 (0.423–0.941)	**0.024**
TTP		
BCLC stage (B/C vs. A)	0.923 (0.530–1.609)	0.779
Tumor encapsulation (complete vs. none)	0.750 (0.541–1.040)	0.085
Tumor size, cm (>5 vs. ≤5)	2.039 (1.270–3.274)	**0.003**
Tumor multiplicity (multiple vs. single)	2.063 (1.399–3.041)	**<0.001**
Vascular invasion (yes vs. no)	1.392 (0.929–2.085)	0.109
TREM2 tumor (positive vs. negative)	0.651 (0.456–0.928)	**0.018**

*BCLC* Barcelona Clinic Liver Cancer, *HR* Hazard ratio, *OS* overall survival, *TTR* time to progression

Bold values indicate *P* < 0.05, *P* values from Cox regression analysis

### TREM2 prevents oncogenic behavior in HCC cell lines

To detect the function of TREM2 in HCC progression, we established stable *TREM2* knockdown and overexpression clones in MHCC97L cells (with a lower lung metastasis rate of 40%) and MHCC97H cells (with a higher lung metastasis rate of 100%), respectively, both of which were established from one parent HCC cell line^23^. As shown by western blotting analysis, downregulation of TREM2 was confirmed in the two TREM2-knockdown clones (KD2 and KD4), and overexpression of TREM2 was also verified in TREM2-overxpression cell line (named TREM2) for subsequent assays (Supplementary Fig. 3).

**Fig. 2:**
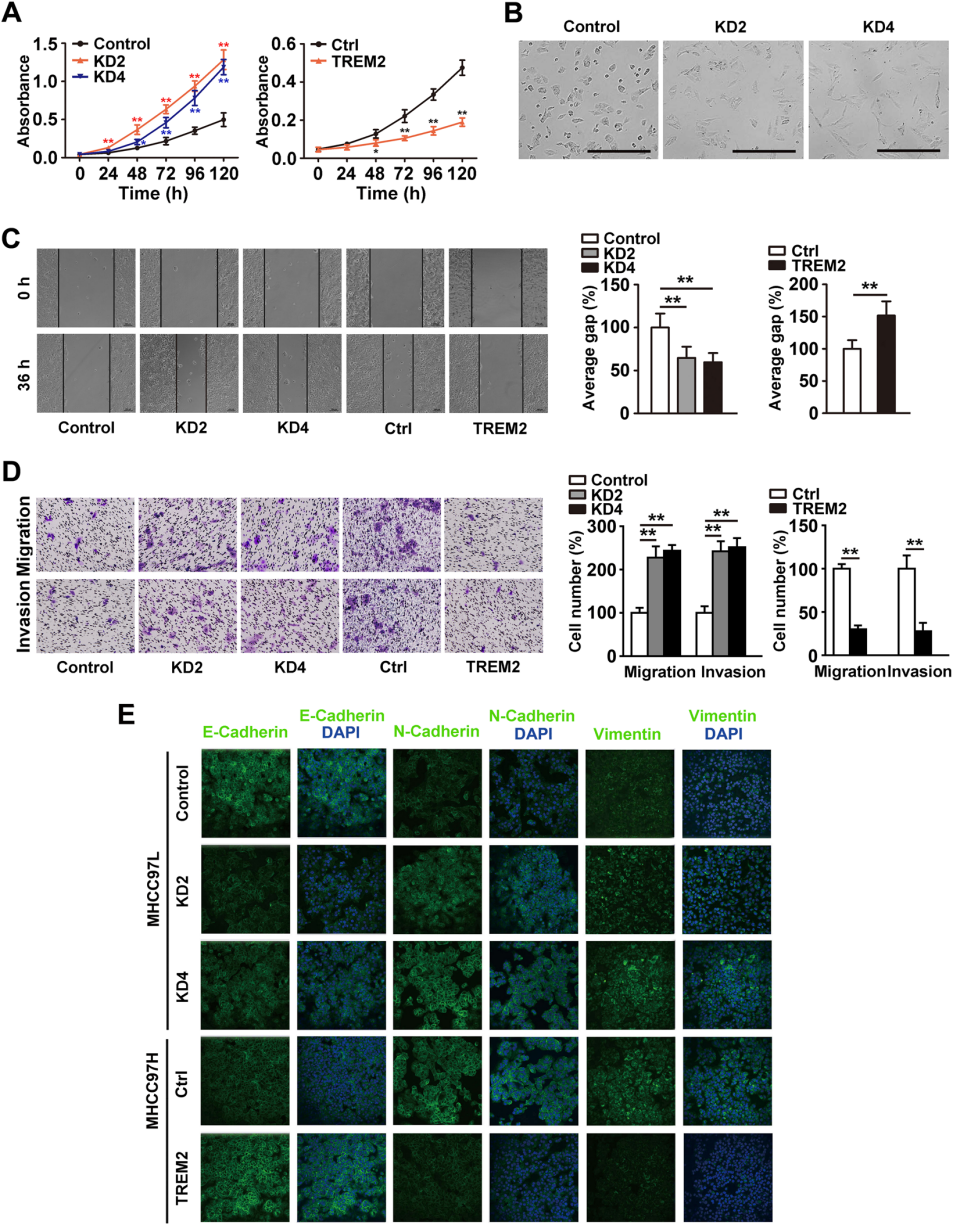
TREM2 reduced oncogenic behaviors in HCC cells. **a** Cell viability was examined by CCK-8 assay. Left panel: The effect of *TREM2* knockdown on cell proliferation ability of hepatoma cells was tested by CCK-8 assay. Right panel: The influence of *TREM2* overexpression on cell proliferation ability of HCC cells showed by CCK-8 assay. **b** Morphological changes in MHCC97H cells with *TREM2* expression. Scale bar, 100 μm. **c** Cell migration was examined using the scratch assay. Left panel: Representative images of the scratch assay in the control MHCC97L and two *TREM2* knockdown cell lines (KD2 and KD4), as well as in the control (Ctrl) MHCC97H and *TREM2* overexpressing cells (TREM2). Right panel: Quantification of the result of the scratch assay. Magnification, ×10. **d** The ability of cell migration and invasion was examined by transwell migration and invasion assays. Left panel: Representative images of transwell cell migration and invasion assay in groups. Right panel: Quantification of the result of transwell assays. Magnification, ×10. **e** Representative images of immunofluorescence staining of EMT-related markers in each group. Data were from a representative experiment carried out in triplicate. *P *<* 0.05, **P *<* 0.01

Compared with the control MHCC97L cells, reduced TREM2 expression markedly promoted HCC cell growth, whereas high TREM2 expression inhibited cell viability (Fig. 2a). Notably, *TREM2* knockdown cell lines became more spindle-like and fibroblastic, along with a longer pseudopodium (Fig. 2b). The suppression of TREM2 expression contributed to a substantial increase in cell motility, migration, and invasion ability of hepatoma cells, while TREM2 stable overexpression produced the opposite effect, as detected by scratch, transwell migration and invasion assays (Fig. 2c, d).

### TREM2 inhibits epithelial to mesenchymal transition (EMT) in hepatoma cells

EMT plays a critical role in tumorigenesis, metastasis, invasion, recurrence, and chemoresistance^24^. We detected the expression level of EMT markers in control and *TREM2* knockdown/overexpression cells. Immunofluorescence staining demonstrated that knockdown of *TREM2* reduced the expression of the epithelial marker E-Cadherin and increased the level of mesenchymal markers N-Cadherin and Vimentin, while overexpression of *TREM2* produced the opposite effect (Fig. 2e). Further western blotting assays verified that TREM2 could suppress EMT (Supplementary Fig. 4a, b). The result of qPCR assay also showed that activation of TREM2 signaling upregulated the mRNA expression levels of E-cadherin, zonula occludens-1 (ZO-1), epithelial cell adhesion molecule (EpCAM), and cytokeratin 18 (CK-18), but downregulated those of matrix metalloproteinase (MMP)-2, MMP-9, snail, and twist (Supplementary Fig. 4c, d).

### The miR-31-5p reduces TREM2 expression in HCC

**Fig. 3:**
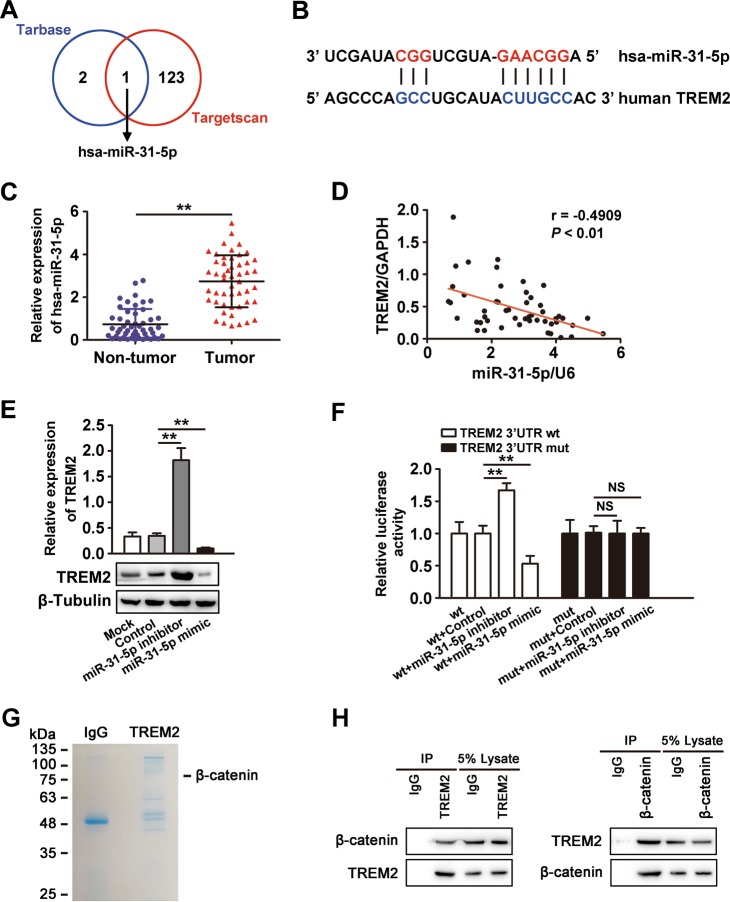
Loss of *TREM2* expression in HCC was regulated by hsa-miR-31-5p. **a** Schema of the search for candidate miRNAs targeting TREM2 using Tarbase and TargetScan. Each labeled circle represented one prediction algorithm with the number of its predicted miRNAs, and hsa-miR-31-5p in the overlapping circles was predicted by both algorithms. **b** miR-31-5p-binding sites in the 3′ UTR of *TREM2* mRNA. **c** The expression pattern of miR-31-5p in 50 cases of tumor tissues and paired non-tumor tissues. **d** Correlation analysis of TREM2 and miR-31-5p according to their expression levels in tumor tissues by qPCR detection. **e** Expression of miR-31-5p could reduce the level of TREM2 while inhibition of miR-31-5p could increase TREM2 expression. TREM2 protein levels were determined after transfection with the indicated miRNA mimic or inhibitor in MHCC97L cells. Quantification of western blotting data was from three separate experiments. **f** Luciferase assay for luciferase reporters with wild-type or mutant TREM2 3′ UTR relative to Renilla luciferase activity in MHCC97L cells transiently transfected with the control, miR-31-5p inhibitor or miR-31-5p mimic. Data were from a representative experiment carried out in triplicate. **g** Proteins that may interact with TREM2 were identified by mass spectrometry. TREM2 protein in *TREM2* overexpressing MHCC97H cells was immunoprecipitated with an anti-TREM2 antibody, separated by SDS-PAGE, stained with Coomassie brilliant blue dye and then subjected to in-gel digestion for mass spectrometry. **h** *TREM2* overexpressing MHCC97H cells were subjected to co-immunoprecipitation with the control IgG, anti-TREM2 or anti-β-catenin antibodies. Loading controls were also indicated

Then we explored the underlying mechanism of decreased TREM2 expression in HCC using 50 cases of tumor and matched non-tumor tissues. First, we considered that *TREM2* gene was not likely to be deleted in the HCC genome, because the result of qPCR showed no difference in the amounts of *TREM2* from genomic DNA between tumor and paired non-tumor tissues (Supplementary Fig. 5a). Second, we have sequenced the coding and untranslated regions of *TREM2* gene in matched non-tumor and tumor tissues, and found no mutations. Third, we found that there is no DNA CpG island in the upstream region of the TREM2-coding region using MethPrimer (Supplementary Fig. 5b). Fourth, we carried out chromatin immunoprecipitation (ChIP) assay and found that histone acetylation of *TREM2* gene was not obviously altered in tumor tissues compared with that in non-tumor tissues (Supplementary Fig. 5c-e). We further searched for potential microRNAs (miRNAs) that could directly bind to the 3′ untranslated region of *TREM2* using two prediction tools Tarbase and TargetScan. One common potential miRNA was selected for further analysis: miR-31-5p (Fig. 3a, b). Then we detected the expression pattern of miR-31-5p in 50 cases of tumor and paired non-tumor tissues by qPCR and found that miR-31-5p expression was higher in tumor tissues than in non-tumor tissues (Fig. 3c). Moreover, the result of correlation analyses showed that miR-31-5p expression was negatively associated with TREM2 expression in HCC tissues (*r* = −0.4909, *P* < 0.01) (Fig. 3d). To further study whether miR-31-5p regulated TREM2 expression, we transfected miR-31-5p inhibitor or mimic into MHCC97L cells. The result implied that the miR-31-5p inhibitor obviously increased *TREM2* expression, whereas the miR-31-5p mimic reduced *TREM2* expression (Fig. 3e). Wild-type and mutant 3′ UTRs of *TREM2* were constructed into a luciferase reporter plasmid, and then co-transfected into MHCC97L cells with miR-31-5p inhibitor, mimic, or the control. The luciferase activity of the wild-type *TREM2* 3′ UTR was substantially increased in the miR-31-5p inhibitor group, while it was markedly reduced in the miR-31-5p mimic group compared with that in the control. No significant difference in the relative luciferase activity was found in different groups with the mutant *TREM2* 3′ UTR, in which the miR-31-5p complementary region within the 3′ UTR of *TREM2* was mutated (Fig. 3f). Thus, the data implied that *TREM2* is a direct target of miR-31-5p in HCC.

### TREM2 could directly interact with β-catenin

We then used liquid chromatography-tandem mass spectrometry (LC-MS/MS) analysis to explore potential proteins that may correlate with TREM2. The differentially immunoprecipitated bands from *TREM2* overexpressing MHCC97H cells were excised from an SDS-PAGE gel and subjected to LC-MS/MS. The analysis revealed that β-catenin might be a TREM2 interacting protein. The result of immunoprecipitation and western blotting verified this observation (Fig. 3g, h).

### The PI3K/Akt/β-catenin signaling pathway is the main pathway responsible for TREM2-mediated oncogenic inhibition

**Fig. 4:**
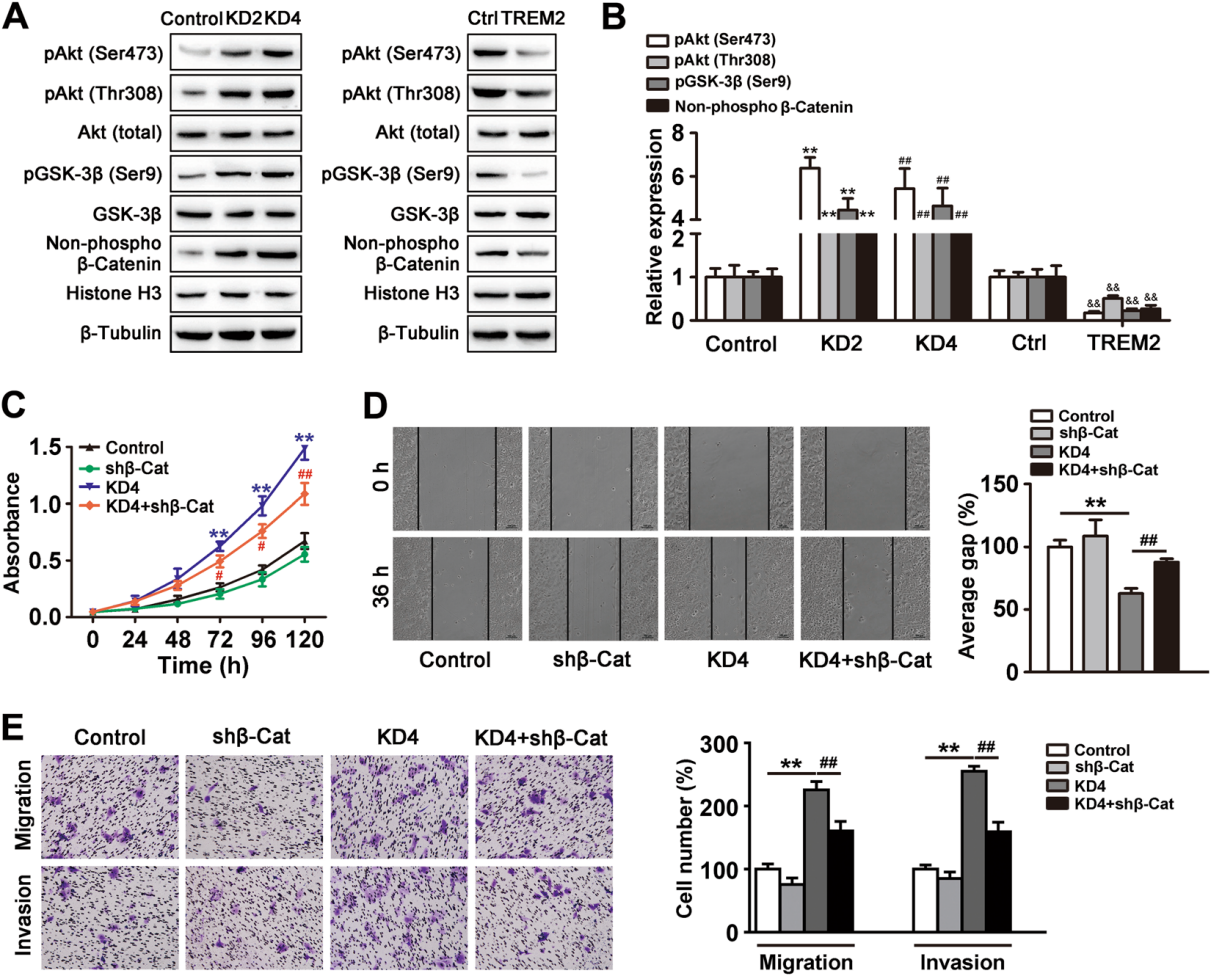
TREM2 influenced tumor behaviors by inhibiting PI3K/Akt/β-catenin signal pathway in vitro and β-catenin interference could rescue the suppressive effect of TREM2 on tumor behavior, such as HCC cell growth, migration, and invasion. **a** Protein level of phosphorylated Akt, phosphorylated GSK3β, and nuclear β-catenin in the control with two *TREM2* knockdown cell lines from MHCC97L and in the control (Ctrl) with *TREM2* overexpressing MHCC97H cells. **b** Quantification of the western blotting data from three separate experiments was shown. *KD2 *vs*. the control, **P *<* 0.01, ^#^KD4 *vs*. the control, ^##^*P* *<* 0.01, and ^&^TREM2 *vs*. the Ctrl, ^&&^*P* *<* 0.01. **c** β-catenin interference significantly rescued *TREM2* knockdown-promoted cell growth as showed by CCK-8 assay. **d** β-catenin interference significantly rescued *TREM2* knockdown-promoted cell motility as showed by scratch assay. Left panel: typical pictures of four groups in scratch assay. Right panel: quantification of the result of scratch assay. **e** β-catenin interference significantly rescued *TREM2* knockdown-promoted migration and invasion of HCC cells as showed by transwell assay. Left panel: Typical pictures of four groups in transwell assay. Right panel: Quantification of the result of transwell assay. Data were from a representative experiment carried out in triplicate. For (**c**–**e**), *TREM2 knockdown *vs*. the control, **P *<* 0.01 and ^#^TREM2 knockdown and β-catenin interference *vs*. TREM2 knockdown, ^#^*P* *<* 0.05, ^##^*P* *<* 0.01

To further evaluate the downstream pathways responsible for the suppressive effect of TREM2 on oncogenic behavior, expression profiles at the mRNA level of *TREM2* overexpressing MHCC97H cells were compared with those of the control by a human liver cancer RT^2^ profile PCR array. We found that a total of seven upregulated (*CDH1*, *OPCML*, *RELN*, *IGFBP1*, *CTNNB1*, *PTEN*, and *TNFSF10*) and four downregulated (*HGF*, *AKT1*, *IRS1*, and *TGFβ1*) genes showed more than a 2.5-fold change in mRNA levels in TREM2 overexpressing cells, compared with those in the control (Table 2). Notably, among 11 differentially expressed genes, PI3K/Akt signaling associated genes including phosphatase and tensin homolog (*PTEN*), *AKT1* and insulin receptor substrate 1 (*IRS1*) were most significantly altered by TREM2 overexpression. To verify this observation, western blotting were carried out to study whether upregulated TREM2 expression in MHCC97H cells could specifically suppress Akt phosphorylation or the PI3K/Akt signaling pathway. TREM2 overexpression induced a marked reduction in the phosphorylation of Akt at Ser473 and Thr308. The results also indicated the substantially increased expression of phosphorylated Akt (Ser473/Thr308) in the *TREM2* knockdown groups (by 6.38-fold/2.4-fold for KD1 and 5.43-fold/3.20-fold for KD2), while they were decreased in the *TREM2* overexpression groups (−5.17-fold/−1.85-fold) (Fig. 4a). A search for the downstream effectors of PI3K/Akt showed that the levels of phosphorylated glycogen synthase kinase 3β (GSK3β) (Ser9) as well as nuclear β-catenin were increased in the *TREM2* knockdown groups (by 4.43-fold/2.25-fold for KD1 and 4.63-fold/1.99-fold for KD2), while they were decreased in the *TREM2* overexpression groups (by −4.48-fold/−4.76-fold). The change in β-catenin distribution by TREM2 appeared to be dependent on PI3K/Akt activation, because the PI3K/Akt inhibitor LY294002 and the PI3K-Akt stimulator IGF-1 abolished the *TREM2* knockdown and *TREM2* overexpression-induced changes in β-catenin distribution, respectively (Supplementary Fig. 6). To address whether β-catenin is involved in TREM2-mediated oncogenic inhibition, we further transfected a β-catenin short-hairpin RNA (shRNA) into the *TREM2* knockdown cells. Beta-catenin interference markedly reversed the influence of *TREM2* knockdown on cell growth, motility, migration, and invasion of HCC cells, indicating that β-catenin is indispensable for TREM2-mediated oncogenic inhibition (Fig. 4c–e). Taken together, the results demonstrated that TREM2 reduced oncogenic behavior via the PI3K/Akt/β-catenin signaling pathway.

**Table 2:** Different gene expression profile between TREM2 overexpression MHCC97H and the control

Gene name	Description	Fold change	*P*	NCBI gene_ID
Increased expression > 2.5-fold				
CDH1	Cadherin 1, type 1, E-cadherin (epithelial)	4.23	<0.01	NM_004360
OPCML	Opioid binding protein/cell adhesion molecule-like	3.87	<0.05	NM_002545
RELN	Reelin	3.38	<0.05	NM_005045
IGFBP1	Insulin-like growth factor binding protein 1	2.72	<0.01	NM_000596
CTNNB1	Catenin (cadherin-associated protein), beta 1	2.66	<0.01	NM_001904
PTEN	Phosphatase and tensin homolog	2.62	<0.05	NM_000314
TNFSF10	Tumor necrosis factor (ligand) superfamily, member 10	2.53	<0.01	NM_003810
Decreased expression > 2.5-fold				
HGF	E74-like factor 3 (ets domain transcription factor, epithelial-specific)	3.76	<0.05	NM_000601
AKT1	V-akt murine thymoma viral oncogene homolog 1	5.71	<0.05	NM_005163
IRS1	Insulin receptor substrate 1	3.17	<0.01	NM_005544
TGFβ1	sex determining region Y-box 15	2.69	<0.05	NM_000660

### TREM2 inhibited tumorigenesis and lung metastasis in vivo

**Fig. 5:**
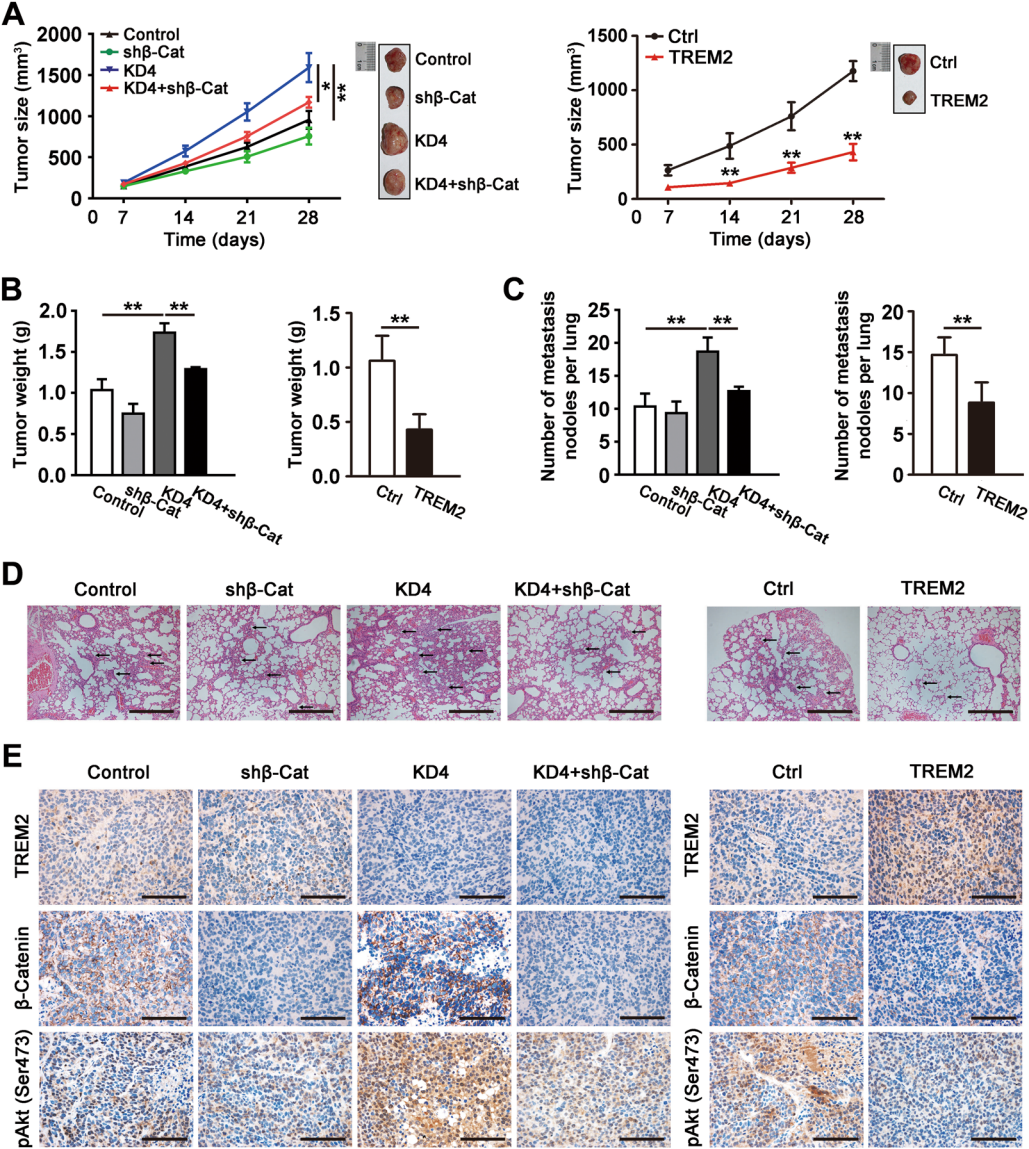
TREM2 prevented tumorigenesis and lung metastasis in a tumor-bearing mouse model and in a lung metastasis model by tail vein injections respectively. **a** *S*ubcutaneous tumor growth curves and representative images of harvested tumors (*n* = 6). **b** Quantification of tumor weight in each group. **c** Quantification of number of tumor foci in six randomly selected high-power fields of the microscope. **d** Typical patterns of lung metastasis in mice (*n* = 6). Microscopic H&E staining of lung tissue samples collected. Scale bar, 200 μm. **e** Typical IHC staining patterns of TREM2, β-catenin, and pAkt in harvested tumors. Scale bar, 50 μm

**Fig. 6:**
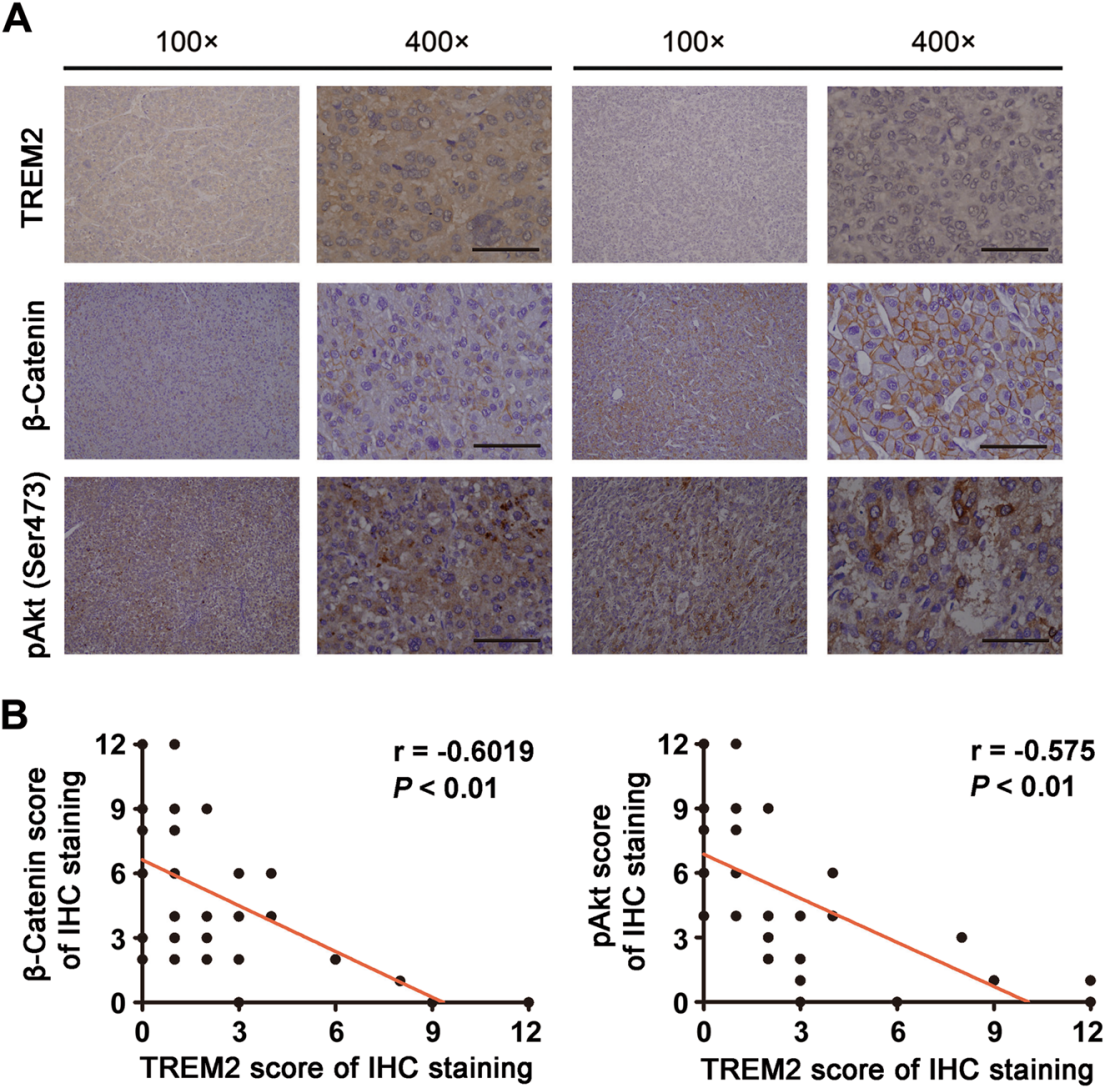
The expression patterns of TREM2, β-catenin, and pAkt in tumor tissues of 50 HCC cases. **a** Typical IHC staining of TREM2, β-catenin, and pAkt in tumor tissues. Scale bar, 50 μm. **b** Correlation analysis between TREM2 and β-catenin scores, along with TREM2 and pAkt scores in tumor tissues

The finding that TREM2 prevented cell growth, migration and invasion in vitro prompted us to explore whether it exerted a similar effect in vivo. A tumor-bearing mouse model was established. The results implied *TREM2* knockdown markedly encouraged tumor growth, while *TREM2* overexpression suppressed tumorigenesis (Fig. 5a, b). Tail vein injection was performed to assess the metastatic potential of hepatoma cells. Histological analysis of lungs confirmed that TREM2 could significantly suppress lung metastasis in mice. The number of tumor foci found in the lungs in the mice injected with *TREM2* knockdown MHCC97L cells was markedly higher than that in the control mice, which could be rescued by β-catenin interference. By contrast, the number of tumor foci in the mice injected with *TREM2* overexpressing MHCC97H cells was less than that in the control. Histological analysis also confirmed that *TREM2* knockdown could promote more lung metastasis compared with that induced by the control cells (Fig. 5c, d). In line with the result of studies in vitro, *TREM2* knockdown significantly upregulated the level of pAkt and activated β-catenin, which could be rescued by β-catenin interference, as indicated by IHC staining. Moreover, *TREM2* overexpression reduced the level of pAkt and activated β-catenin in vivo (Fig. 5e). To further investigate the correlation between TREM2 expression level and the PI3K/Akt/β-catenin signaling in HCC, immunohistochemistry was performed to analyze the staining patterns of TREM2, pAkt, and activated β-catenin in tumor tissues of 50 HCC patients. Correlation analysis indicated that TREM2 level was negatively associated with pAkt (*r* = −0.6019, *P* < 0.01) and activated β-catenin (*r* = −0.575, *P* < 0.01) (Fig. 6).

### TREM2 expression in other gastrointestinal tumors

In addition, we explored TREM2 expression patterns in other gastrointestinal tumors. We found similarly reduced level of TREM2 in tumor compared with that in non-tumor tissues in gastric cancer, while there was increased expression in tumor tissues of pancreatic cancer. However, there existed no difference in TREM2 level between tumor and matched non-tumor tissues in colorectal cancer or esophageal squamous cell carcinoma (Supplementary Fig. 7).

## Discussion

As a novel pattern recognition receptor family, TREMs have recently emerged as critical immune regulators that modulate the inflammatory response. TREM signaling relies on its interaction with DAP12, a cytosolic adapter that could associates with a variety of corresponding receptors, leading to an excitatory or inhibitory inflammatory response^25^. Previous studies have revealed that TREM2 was engaged in the negative regulation of inflammatory and immune responses^9,15^. Silencing of *TREM2* enhanced the inflammatory responses of alveolar macrophages to LPS^26^. In a mice model of LPS-induced acute lung injury, TREM2 was substantially decreased in lung tissues^27^. Although TREM2 has been studied in nonmalignant disease, there are few reports on its role in malignant diseases. A recent study showed that TREM2 negatively regulated immune response via Syk signal pathway in an IL-10 dependent manner in lung cancer^28^. Upregulation of TREM2 enhanced capacity of proliferation and invasion in glioma cells^29^. Herein, we investigated TREM2 expression in tumor and matched non-tumor tissues of HCC patients. We observed that TREM2 levels correlated with pathological characteristics including BCLC stage, tumor size, tumor encapsulation, vascular invasion and tumor differentiation, suggesting that TREM2 may be associated with HCC progression. Survival analysis showed that TREM2 deficiency in tumor tissues was associated with a shorter survival and higher risk of recurrence in HCC patients. TREM2 expression in tumor tissues was found to be an independent prognostic factor of survival and recurrence. These data revealed a previously unknown role of TREM2 in HCC progression.

After demonstrating that TREM2 downregulation occurred frequently in HCC and correlated significantly with the invasive features of HCC, we explored the effect of TREM2 on HCC development by assays in vitro and in vivo. In the present study, two clones with relatively low and high metastatic potential (i.e., MHCC97L and MHCC97H, respectively) isolated from one parent cell line^23^ were used to better explore the tumor biological effect of TREM2 on HCC progression. The results showed that *TREM2* knockdown greatly promoted tumor behaviors including cell growth, migration, and invasion in vitro, which was then verified in vivo using a mouse tumor-bearing model and lung metastasis model via tail vein injections in nude mice, while *TREM2* overexpression showed the opposite effect. Therefore, we hypothesized that TREM2 is closely related to HCC tumorigenesis and metastasis. After that we explored the upstream regulation mechanism of decreased *TREM2* expression in HCC tumor tissues. By target-prediction tools, we found that miR-31-5p may target *TREM2* expression. The result of qPCR indicated the increased expression profile of miR-31-5p in HCC tissues, which was negatively associated with *TREM2* expression. Further luciferase reporter assay in vitro verified that *TREM2* was a direct target of miR-31-5p in HCC.

The analysis of immunoprecipitation and mass spectrometry data showed that TREM2 could directly interact with β-catenin. As an important downstream protein of the PI3K/Akt pathway, β-catenin plays a vital role in HCC metastasis and carcinogenesis^30^. Abnormal β-catenin accumulation has been verified in 17–40% of HCC. The low expression level of β-catenin in hepatocytes is attributed to the activity of a destruction complex comprising adenomatous polyposis coli, AXIN, and two kinases, GSK3 and casein kinase α/β, which phosphorylate β-catenin thus promoting its degradation via the proteasome. When the β-catenin destruction complex is dissolved, β-catenin subsequently accumulates in the cytoplasm. Then β-catenin could translocate to the nucleus where it serves as a transcription activator and forms a transcriptional complex with the T-cell factor protein, thus activating the downstream target genes which accelerate cell proliferation, cell cycle, cell migration, and invasion^31^. Activation of β-catenin by hypoxia in hepatoma cells leads to enhanced metastatic potential and poor prognosis^32^. In the present study, *TREM2* knockdown significantly increased the level of active β-catenin (non-phosphorylated β-catenin). We speculated that TREM2 may inhibit β-catenin by interacting with it, thus causing exposure of corresponding phosphorylation sites of β-catenin, or enhancing the coherence of the β-catenin destruction complex, which requires further in-depth study.

**Fig. 7:**
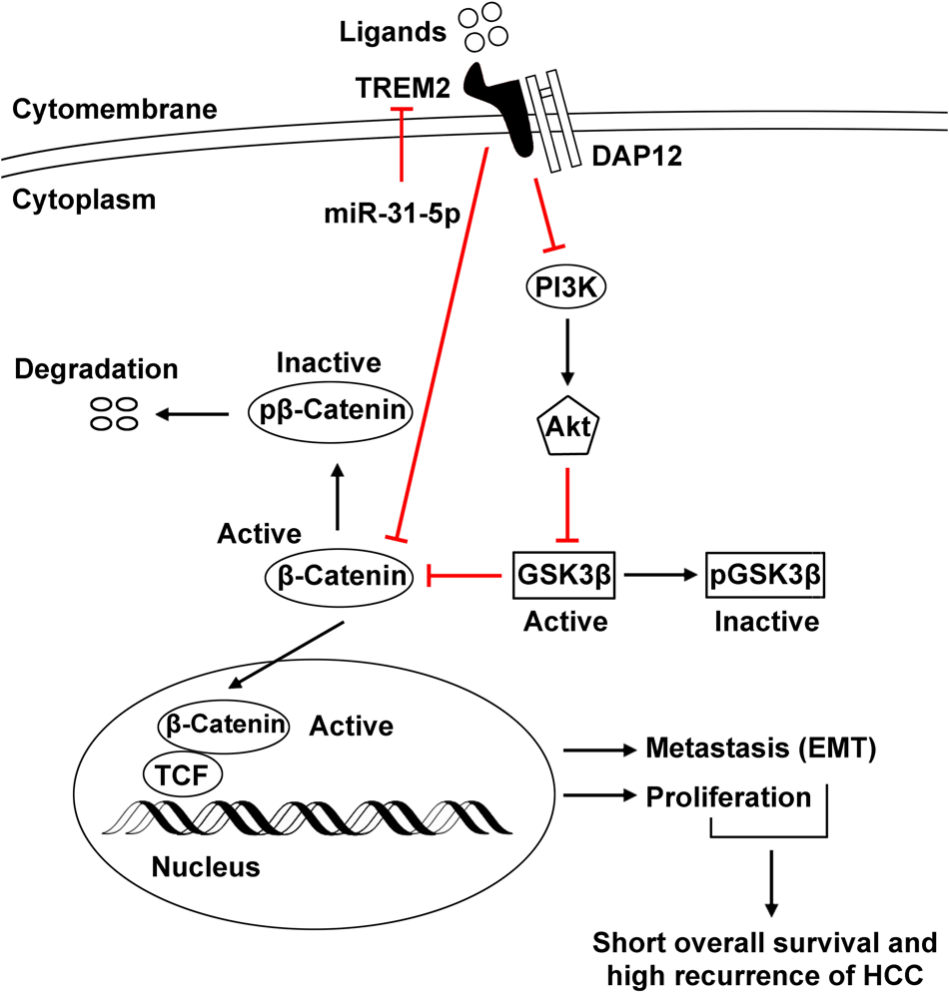
Schematic diagram depicting the role of TREM2 in HCC progression and metastasis. Loss of *TREM2* in HCC tumor tissues was regulated by miR-31-5p. TREM2 could interact with β-catenin. Moreover, TREM2 inhibited the phosphorylation of Akt and GSK-3β, thus suppressing β-catenin accumulation in the cytosol and translocation to the nucleus

A further mechanistic study based on a PCR array revealed that TREM2 may exert a tumor inhibitory role in HCC through the PI3K/Akt signal pathway. The PI3K/Akt signaling network broadly regulates divergent physiological processes, including apoptosis, cell cycle, differentiation, progression, transcription, and translation, and is closely correlated to cancer tumorigenesis and metastasis^33^. Peng et al. reported PI3K is recruited to the TREM2/DAP12 signal complex upon the interaction between regulatory subunit p85 and the adaptor protein DAP12 in mice bone marrow macrophages^34^. TREM2 promotes host resistance against *Pseudomonas aeruginosa* infection as well as macrophage-mediated eradication of *P. aeruginosa* by activating the PI3K/Akt signaling in murine macrophages^35,36^. The above study suggested the involvement of PI3K in the downstream TREM2 signaling pathway in immunity. Next, we explored the influence of TREM2 on the PI3K/Akt pathway in HCC. Notably, we found that *TREM2* knockdown could enhance the phosphorylation of Akt and GSK3β and activated β-catenin in HCC cells, while the reverse was observed in TREM2 overexpressing cells, which were the opposite results to those of reported by previous studies in other cell types. We speculated that different condition of cells and cell types may account for the phenomenon. In normal cells, the corresponding signal pathways are in a normal state. However, in cancer cells, the signal pathways are aberrant and disorganized. Further rescue assays in vitro and in vivo indicated that the tumor-promoting effect of *TREM2* knockdown could be rescued by β-catenin interference. Based on these observations, we hypothesized that TREM2 suppress HCC metastasis and development by targeting the PI3K/Akt/β-catenin pathway (Fig. 7). However, whether the interaction of TREM2 with β-catenin or the inhibitory effect on PI3K signaling of TREM plays a more important role in HCC development remains unknown. In addition, why TREM2 and beta-catenin interact requires further study. Moreover, our study revealed that TREM2 could suppress EMT. We also detected the *TREM2* expression patterns in other gastrointestinal tumors and found various expression patterns of TREM2. We concluded that *TREM2* expression exhibits tissue specificity and cell specificity.

In conclusion, our study identified TREM2 as a tumor suppressor in HCC. Additionally, we verified that TREM2 suppressed HCC metastasis via the PI3K/Akt/β-catenin pathway. Therefore, TREM2 could be a prospective marker and therapeutic target in HCC.

## Materials and methods

### Patients and specimens

HCC specimens from two cohorts of 250 and 135 patients who underwent surgical resection between 2009 and 2010 were obtained from Zhongshan Hospita of Fudan University. Ethical approval for the study was obtained from Zhongshan Hospital Ethics Committee and informed consent was obtained from each patient. Fresh tumor, matched non-tumor liver tissues and venous metastasis were collected for analysis at mRNA or protein level. All subjects were contacted every 3 months during the first postoperative year, and at least 6 months afterward for survival and recurrence inquiry until death, contact failure, or until the end of the investigation. Patients were monitored prospectively by serum AFP and ultrasound examination every 3–6 months. A diagnosis of recurrence was determined based on a typical imaging appearance in ultrasound or liver biopsy.

### Tissue microarrays and immunohistochemical staining

TMAs were constructed by Shanghai Biochip Company (China). Core samples were obtained from representative regions from each tumor on hematoxylin and eosin staining. Two 1.5 mm cores were taken from the tissue block for each case (tumor tissue and paired non-tumor liver tissue). Tissue microarrays were constructed using an arraying machine (Beecher Instruments).

Paraffin-embedded sections were deparaffinized and subjected to antigen retrieval with citrate buffer (pH = 6.0). Sections were then incubated overnight at 4 °C with primary antibody. After incubated with secondary antibody for 30 min, sections were visualized with 3, 3′-diaminobenzidine solution and counterstained with hematoxylin. The primary antibodies are listed in the Supplementary Information. The sections were observed under a light microscopy to examine tumor microheterogeneity in antigen distribution. A semi-quantitative scoring system for assessing protein expression was based on the staining intensity (0, negative; 1, weak; 2, intermediate; 3, strong) and the percentage of positive cells (0, 0% positive cells; 1, 1–25% positive cells; 2, 26–50% positive cells; 3, 51–75% positive cells; 4, 76–100% positive cells)^37^. The composite expression score (CES) was obtained by multiplying the scores of staining intensity and percentage of positive cells, with full range from 0 to 12. CES ≤ 4 was considered as low and negative TREM2 expression and CES > 4 as high and positive expression according to receiver operating characteristic analysis (Supplementary Fig. 1). The evaluation of IHC staining was performed by two independent pathologists who were unaware of the patient outcomes.

### Immunoprecipitation and mass spectrometry

Cells were lysed with immunoprecipitation lysis buffer, which was constituted of 50 mM Tris-HCl, pH 7.5, 150 mM NaCl, 0.1% Nonidet P-40, 5 mM EDTA, 5 mM EGTA, 15 mM MgCl_2_, 60 mM beta-glycerophosphate, 0.1 mM sodium orthova-nadate, 0.1 mM NaF, 0.1 mM benzamide, 10 mg/ml aprotinin, 10 mg/ml leupeptin, 10 mg/ml phenylmethylsulfonyl fluoride. The precleared lysates were incubated with primary antibody for 1 h at 4 °C and then with protein A/G agarose beads overnight at 4 °C. The bound proteins were eluted by boiling in lysis buffer and then tested by western blotting. For peptide analyses, after immunoprecipitation, bands corresponding to TREM2 were cut out and gel purified, digested using chemotrypsin and analyzed by LC-MS/MS.

### Animal models

Four-week-old male nude mice were purchased from Shanghai Slac Laboratory Animal Company and were raised in specific pathogen-free conditions. To assess the tumorigenicity of cells, tumor-bearing mice model were conducted as described^38^. Cells were trypsinized and resuspended in a 50% mixture of Matrigel (BD Biosciences) in DMEM and then to injected in the right flank of each mouse with 1 × 10^7^ cells. To examine the metastatic ability of cells, lung metastasis model were conducted. Lung metastasis model were constructed by injecting 1 × 10^6^ cells via the tail vein. Mice were sacrificed at 4 weeks post-injection. Tumor tissues were collected, photographed and weighted. Consecutive tissue sections (110–135 sections) were prepared for each lung, and stained with haematoxylin-eosin^39^. Experimental procedures were complied with guidelines established by Shanghai Medical Experimental Animal Care Commission.

### Statistical analysis

Statistical analyses were performed by SPSS 19.0 software. Categorical variables were compared by Chi-square test and continuous variables were compared using *t* test. Variables related to OS and TTR were verified using univariable Cox proportional hazards regression models. Significant factors in univariable analysis were further tested in a multivariable Cox regression analysis in a stepwise manner. Survival curves were generated by Kaplan–Meier plots (log-rank tests). The results were presented as mean ± standard deviation from three independent experiments. The association between *TREM2* expression and miR-31-5p or pAkt or β-catenin was examined by Pearson correlation analysis. A two-tailed *P* < 0.05 was considered statistically significant.

## Supplementary information

Supplementary information

Supplementary figure 1

Supplementary figure 2

Supplementary figure 3

Supplementary figure 4

Supplementary figure 5

Supplementary figure 6

Supplementary figure 7
