# Supplementary material for: TREM2 acts as a tumor suppressor in hepatocellular carcinoma by targeting the PI3K/Akt/β-catenin pathway
Source: Oncogenesis. 2019 Jan 25;8(2):9. doi: 10.1038/s41389-018-0115-x (PMC6350080; doi:10.1038/s41389-018-0115-x)
Supplement: Supplementary file 1 — Supplementary information [file 41389_2018_115_MOESM1_ESM.doc]

**Supplementary Materials and Methods**

**Antibodies and reagents**

PI3K inhibitor LY294002 as well as EMT Antibody sampler kit (#9782) including Vimentin, N-Cadherin, ZO-1, Snail, Slug and E-Cadherin were purchased from Cell Signaling Technology. Histone H3 (sc-10809) and β-Tubulin (sc-9104) were purchased from Santa Cruz Biotechnology. Βeta-catenin (ab32572), TREM2 antibody (ab86491) for western blotting, TREM2 antibody (ab85851) for immnohistochemistry and TREM2 antibody (ab125117) for immunoprecipitation were purchased from Abcam. Recombinant human IGF-1 were purchased from R&D Systems. Has-miR-31-5p inhibitor (MH11465) and mimic (MC11465) were purchased from Thermo Fisher Scientific.

**Cell lines and transfection**

Human hepatoma cell lines (Hep3B, Huh7, and PLC/PRF/5) and THLE-3 were purchased from the cell bank of Chinese Academy of Sciences (Shanghai, China). [HCCLM3](http://www.google.com.hk/url?sa=t&rct=j&q=MHCC-LM3&source=web&cd=1&cad=rja&ved=0CDQQFjAA&url=http%3A%2F%2Fwww.biogou.com%2Fgoods.php%3Fid%3D142226&ei=aAhAUb3hHtSeiQfqtIC4BQ&usg=AFQjCNFjCxhMhQ4Vk-316yBdSl4kqP_TOA), MHCC97H and MHCC97L were established in Liver Cancer Institute of Zhongshan Hospital, Fudan University. Hepatoma cells were maintained in DMEM (Invitrogen) supplemented with 10% FBS (GIBICO). THLE-3 was maintained in BEGM (Walkersville, MD) containing 10% FBS. All cell lines have been authenticated by STR analysis during the study period and have been tested for mycoplasma contamination.

Transfection assays were conducted using Lipofectamine 3000 (Thermo Fisher) according to the manufacturer's protocol. Beta-catenin shRNA (TRCN0000003843) were obtained from Sigma Aldrich. The experiment was repeated three times.

**Construction of TREM2 knockdown and overexpression clones**

Five short hairpin RNA targeting TREM2 (nominate KD 1~5), non-mammalian shRNA control and TREM2 in pCDH vector plasmid was obtained from Jinshengte Corporation (China). The corresponding lentivirus was produced by Jinshengte Corporation. Cells at a confluence of 60% in 6-well plates were infected with lentivirus, selected by 2 μg/ml puromycin for 1 week and then the protein level was detected by western blotting.

**Western blotting**

Liver tissue or harvested cells were lysed in RIPA buffer (Beytime). Protein concentrations were determined by BCA Protein Assay Kit (Pierce). Forty micrograms of protein were resolved on 10% SDS-PAGE, transferred to polyvinylidene difluoride membranes, blocked with 5% non-fat dry milk for 1 hour at room temperature and then incubated with primary antibodies at 4°C overnight. Blots were detected by ECL reagent (Tiangen). Quantification of western blotting data was from three separate experiments.

**Cell function assays *in vitro***

In cell viability assay, Cell Counting Kit-8 (CCK-8, Dojindo) was done to evaluate cell viability and growth. Cells were seeded in 96-well plate at a confluence of 10%. After incubating cells with CCK-8 at 37°C for 60 min, cell proliferation rate was tested by reading absorbance at 450 nm at the indicated time points according to the manufacturer’s instructions.

Scratch assay was conducted to detect the capacity of cell migration and growth. Cells were grown to 90-95% confluence in 6-well plates, and a wound was generated by scratching into the cell monolayer with a 200 μl pipette tip. Cells were then incubated with medium free of FBS for 36 h, and photographed by a microscope.

Transwell migration and invasion assay was conducted in chamber of 8 μm-pore Transwell inserts. For cell migration assay, 2×10^4^ cells were seeded into the top chamber of each insert in serum-free medium and 10% serum-containing medium was used in the lower chamber. For cell invasion assay, 1×10^5^ cells were seeded. Transwell inserts were precoated with 100 μl of Matrigel (BD biosciences) and was solidified at 37°C for 12 h. Cells that had migrated or invaded were fixed by 4% paraformaldehyde and stained with the crystal violet for image capture and counted under a light microscope.

Data are representative of three independent experiments performed in triplicate.

**PCR arrays and quantitative PCR (qPCR)**

For detecting the expression profile of genes in particular pathways, liver cancer RT^2^ Profiler PCR arrays (SABiosciences, Qiagen, USA) were used. Data were analyzed by instrument’s software. Data were normalized for GAPDH levels by the ΔΔCt method. Total RNA was extracted from cells using TRIzol reagent (Invitrogen) according to the manufacturer’s instruction. For qPCR of genes, total RNA was reversed to cDNA by PrimeScrip RT Master Mix (Takara) and the mRNA expressions was detected using SYBR Premix Ex Taq II (Takara). The primers synthesized by Shanghai Shenggong biocompany were listed in Supplementary Table 5. For qPCR of miRNA, specific miRNA primers for the reverse transcriptions of hsa-miR-31-5p (A25576, Thermo Fisher Scientific) and the internal control U6 (001973, Thermo Fisher Scientific) were used. TaqMan microRNA Reverse Transcription kit (Applied Biosystem) were used according to the manufacturer’s instructions. Quantitative real-time PCR was performed in a StepOne Plus Realtime PCR system (Applied Biosystems). The experiment was repeated three times with different sets of samples.

**Immunofluorescence**

Cells seeded on glass coverslips were fixed with 4% paraformaldehyde at room temperature for 30 min, washed 3 times with PBS, permeabilized with 0.2% Triton X-100, and blocked for 1 hour at room temperature with PBS containing 5% BSA. Afterwards, cells were incubated with primary antibody overnight at 4°C. The next day the cells were incubated with Alexa Fluor 488-conjugated secondary antibody for an hour at room temperature. Stained cells were observed under a confocal fluorescence microscopy (Nikon).

**Mutation analysis**

Genomic DNA (gDNA) was extracted from matched non-tumor and tumor tissues using the TIANamp Genomic DNA kit (Tiangen) following the manufacturer's protocol. Primers flanking all coding exons and intron-exon boundaries of the gene TREM2 gene were amplified by PCR using primer sets following: exon 1, 5’-CCCAGTCCTGACTATTGCTT-3’ and 5’- GCCACCGCCTTCATAATTCA -3’; exon 2, 5’-GCTCCTTCAGGGCAGGATTT-3’ and 5’- AGTGGGTGGTTCTGCACAC-3’; exon 3, 5’-AGTGTAATGACCTGATCCACATAGGA-3’ and 5’-GCTCTAGTTGCCTTGTAATTTGTAGT-3’; and exons 4 and 5, 5’- GCAGGTTAGTGGCAGAGCAT-3’ and 5’-GAAGCAGTGTTCAGGCAGAG-3’. The purified PCR products were processed for direct sequencing on the 3730xl DNA Analyzer (Applied Biosystems, Foster City, CA, USA). The gDNA samples were subsequently amplified through PCR, and PCR products were purified and directly sequenced using an ABI PRISM 3730 automated sequencer (Applied Biosystems).

**CpG island prediction**

MethPrimer (http://www.urogene.org/methprimer/) were used to search for the CpG islands of TREM2 gene^1^. The criteria used was island size > 100, GC Percent > 50.0, and observed-to-expected ratio (Obs/Exp) > 0.6. The prediction results showed there were no CpG islands in *TREM2* sequence at transcriptional start sites (from upstream -2000 to downstream 1000).

**ChIP assay**

ChIP was conducted in tissue homogenates as previously described^2^. In brief, chromatin was prepared from 100 mg of tissue. Proteins and DNA were reversibly cross-linked with 1% formaldehyde for 10 min at 37℃. DNA was sheared by sonication on ice, yielding 0.5-1 kb fragments of DNA. Chromatin was incubated with anti-acetyl-histone H3 (06599, Upstate), anti-acetyl-histone H4 (06598, Upstate), and non-specific IgG (2729, CST) or no antibody. After immunoprecipitation, DNA-protein crosslinks were reversed with proteinase K, and DNA was isolated using phenol chloroform extraction. Relative amounts of DNA was detected by qPCR. Sequence specific PCR primer sets for specific regions (within −3000 to +1000 region, corresponding to the transcription start sites of genes) of human TREM2 genes were showed in Supplementary Table 6. The data obtained were normalized to the corresponding DNA input control.

**In silico prediction of miRNAs targeting the 3′-UTR of TREM2**

Two algorithms were used to predict potential miRNAs targeting TREM2's 3′-UTR: [TarBase v.8](http://carolina.imis.athena-innovation.gr/diana_tools/web/index.php?r=tarbasev8%2Findex)(http://carolina.imis.athena-innovation.gr/diana_tools/web/index.php?r=tarbasev8%2Findex/) and Targetscan (<http://www.targetscan.org/>).

**Luciferase reporter assay**
The wild-type with the putative miR-31-5p binding site in the 3’UTR of TREM2 and its mutant dual-luciferase reporter vectors were constructed by Hanyin corporation (Shanghai, China). The 3’UTR of human *TREM2* gene was amplified by PCR using primer set (forward primer: 5’- CCGCTCGAGAGGAAGATGATGGGAGGAAAAGCC-3’; reverse primer: 5’-ATAAGAAT GCGGCCGCTTATCCAGCTAAATATGACAGTCTTGGAT-3’). The PCR product was cloned into the multiple cloning site located downstream of the luciferase reporter gene in the dual-luciferase reporter vector pmiGLO basic between XhoI and NOT I site to generate the pmirGLO-TREM2-WT. And the mutant vector was amplified using primers (forward primer 1: 5’-CCGCTCGAGAGGAAGATGATGGGAGGAAAAGCC-3’; reverse primer 1: 5’-GCCAAGTTATCCTTATGCAGGCTGGGCTGGTCC-3’; forward primer 2: 5’-CTGCATAAGGATAACTTGGCCACCAGGACTCCT-3’; reverse primer 2: 5’- ATAAGAAT GCGGCCGCTTATCCAGCTAAATATGACAGTCTTGGAT -3’). The PCR product was inserted into the pmirGLO basic. The experiment was repeated three times with similar results.

**References**

1. Li, L.C. & Dahiya, R. MethPrimer: designing primers for methylation PCRs. *Bioinformatics*. **18**, 1427-1431 (2002).

2. Braveman, M.W., Chen-Plotkin, A.S., Yohrling, G.J. & Cha, J.H. Chromatin immunoprecipitation technique for study of transcriptional dysregulation in intact mouse brain. *Methods Mol Biol*. **277**, 261-276 (2004).

**Supplementary Figure Legends**

**Supplementary Fig. 1** Receiver operating characteristic (ROC) curve analyses of IHC staining of TREM2. The result indicated the optimal cut-off value of composite expression score was 4 (CES4), and the area under the ROC curve is 0.636 (95% CI, 0.521-0.752, *P* < 0.05). CES > 4 represented TREM2 high and positive expression and CES ≤ 4 represented TREM2 low and negative expression.

**Supplementary Fig. 2** Kaplan-Meier curve of overall survival (OS) and time to progression (TTP) for expression of *TREM2* in a different HCC dataset comprised of 135 HCC patents. **a** Kaplan-Meier curve of OS for *TREM2* expression. **b** Kaplan-Meier curve of TTP for *TREM2* expression.

**Supplementary Fig. 3** Efficiency of *TREM2* knockdown and overexpression was examined by western blotting. **a** Protein level of TREM2 in the control group and five knockdown cell lines (KD1-5). KD2 and KD4 revealed efficient knockdown of *TREM2* expression. **b** Expression of TREM2 in the control and *TREM2* overexpression cells. Relative intensity of TREM2 normalized to β-Tubulin was calculated. Quantification of western blotting data was from three separate experiments. ***P* < 0.01.

**Supplementary Fig. 4** TREM2 inhibited EMT in hepatoma cells and could interact with β-catenin. **a** Protein level of epithelial markers including E-Cadherin, Claudin-1 and ZO-1 as well as mesenchymal markers including N-Cadherin and Vimentin in control and *TREM2* knockdown/overexpression groups. **b** Quantification of expression levels of EMT related proteins. **c** Expression of epithelial markers (E-Cadherin, ZO-1, EpCAM, CK-18 and CK-19) at mRNA level. **d** Expression of mesenchymal markers (MMP-2, MMP-9, Snail, Slug and Twist) at mRNA level. Quantification of western blotting and qPCR data was from three separate experiments. ***P* < 0.01; NS, no significance.

**Supplementary Fig. 5** The reduced expression of TREM2 was not regulated by deletion, methylation and acetylation. **a** The amounts of *TREM2* from genomic DNA of tumor and paired non-tumor tissues was measured by qPCR. **b** CpG island prediction was performed using MethPrimer. CpG islands were not found in TREM2 sequence at transcriptional start sites. **c** Schematic of the location of primer sets which were spaced at 500-1000 bp intervals. *TREM2* is located 104 bp upstream of the translation initiation site. The arrow indicated the transcription initiation sites. **d** The H3 acetylation pattern was evaluated using ChIP. **e** The H4 acetylation pattern was tested by ChIP. NS, no significance.

**Supplementary Fig. 6** TREM2 influenced tumor behaviors by inhibiting PI3K/Akt/β-catenin signal pathway *in vitro*. **a** Protein level of phosphorylated Akt, phosphorylated GSK3β, and nuclear β-catenin in MHCC97L cells pretreated with 25 μΜ LY294002 for 24 h and in MHCC97H cells pre-administrated with100 ng/ml IGF-1 for 30 min. **b** Quantification of the western blotting data from three separate experiments was shown. NS, no significance.

**Supplementary Fig. 7** TREM2 expression in other gastrointestinal tumors. There was reduced expression of TREM2 in tumor tissues of gastric cancer and increased expression in non-tumorous tissues of pancreatic cancer compared to the non-tumorous tissues. However, there existed no difference of TREM2 level between tumor and non-tumor liver tissues in colorectal cancer as well as esophageal squamous cell carcinoma.

**Supplementary Tables**

Supplementary Table 1. Clinicopathologic features of human tissue specimens enrolled in the study.

| Patients | 250 |
| --- | --- |
| Gender |  |
| Female | 31 |
| Male | 219 |
| Age, years |  |
| ≤52 | 94 |
| >52 | 156 |
| Hepatitis B surface antigen |  |
| Negative | 26 |
| Positive | 224 |
| ALT (units/L) |  |
| ≤75 | 225 |
| >75 | 25 |
| AFP (ng/mL) |  |
| ≤20 | 104 |
| >20 | 146 |
| Liver cirrhosis |  |
| No | 36 |
| Yes | 214 |
| BCLC stage |  |
| A | 113 |
| B/C | 137 |
| Tumor size (cm) |  |
| ≤5 | 130 |
| >5 | 120 |
| Tumor multiplicity |  |
| Single | 207 |
| Multiple | 43 |
| Tumor encapsulation |  |
| No | 124 |
| Complete | 126 |
| Vascular invasion |  |
| No | 165 |
| Yes | 85 |
| Tumor differentiation |  |
| I-II | 198 |
| III-IV | 52 |

Abbreviations: AFP, [alpha fetoprotein](http://www.iciba.com/alpha_fetoprotein); ALT, alanine aminotransferase; BCLC, Barcelona Clinic Liver Cancer.

1

Supplementary Table 2. Correlation between TREM2 and clinicopathologic characteristics.

|  | TREM2 expression in tumor tissue | | |
| --- | --- | --- | --- |
| Clinical indexes | Negative | Positive | *P* value |
| Patients | 163 | 87 |  |
| Gender |  |  |  |
| Female | 18 | 13 | 0.373 |
| Male | 145 | 74 |  |
| Age (years) |  |  |  |
| ≤52 | 62 | 32 | 0.845 |
| >52 | 101 | 55 |  |
| Hepatitis B surface antigen |  |  |  |
| Negative | 16 | 10 | 0.679 |
| Positive | 147 | 77 |  |
| ALT (units/L) |  |  |  |
| ≤75 | 149 | 76 | 0.309 |
| >75 | 14 | 11 |  |
| AFP, ng/mL |  |  |  |
| ≤20 | 66 | 38 | 0.626 |
| >20 | 97 | 49 |  |
| Liver cirrhosis |  |  |  |
| No | 20 | 16 | 0.189 |
| Yes | 143 | 71 |  |
| BCLC stage |  |  |  |
| A | 64 | 49 | **0.010** |
| B/C | 99 | 38 |  |
| Tumor size (cm) |  |  |  |
| ≤5 | 73 | 57 | **0.002** |
| >5 | 90 | 30 |  |
| Tumor multiplicity |  |  |  |
| Single | 132 | 74 | 0.420 |
| Multiple | 31 | 13 |  |
| Tumor encapsulation |  |  |  |
| No | 89 | 35 | **0.030** |
| Complete | 74 | 52 |  |
| Vascular invasion |  |  |  |
| No | 100 | 66 | **0.021** |
| Yes | 63 | 21 |  |
| Tumor differentiation |  |  |  |
| I-II | 123 | 75 | **0.046** |
| III-IV | 40 | 12 |  |
| Abbreviations: AFP, [alpha fetoprotein](http://www.iciba.com/alpha_fetoprotein); ALT, alanine aminotransferase; BCLC, Barcelona Clinic Liver Cancer. Bold values indicate *P* < 0.05, *P* values from χ^2^ test. | | | |

Supplementary Table 3. Univariate analyses of factors correlated with overall survival (OS) and time to progression (TTR) of HCC pateints.

|  | Variables | OS | | TTP | |  |
| --- | --- | --- | --- | --- | --- | --- |
|  |  | HR (95％CI) | *P* | HR (95％CI) | *P* |  |
|  | Gender (male vs. female) | 0.915  (0.557-1.503) | 0.726 | 1.197 (0.733-1.955) | 0.473 |  |
|  | Age, years (>52 vs. ≤52) | 0.834 (0.594-1.173) | 0.297 | 0.821 (0.597-1.128) | 0.223 |  |
|  | Hepatitis B surface antigen (positive vs. negative) | 0.901  (0.535-1.517) | 0.695 | 0.840 (0.514-1.372） | 0.485 |  |
|  | BCLC stage (B/C vs. A) | 1.697 (1.198-2.404） | **0.003** | 1.912  (1.388-2.632) | **<0.001** |  |
|  | Liver cirrhosis (yes vs. no) | 1.209  (0.736-1.985) | 0.453 | 0.889  (0.566-1.395) | 0.609 |  |
|  | ALT, units/L (>75 vs. ≤75) | 1.326 (0.775-2.268 | 0.303 | 1.176 (0.701-1.972) | 0.538 |  |
|  | AFP, ng/mL (>20 vs. ≤20) | 1.148 (0.818-1.613) | 0.424 | 1.125 (0.822-1.541) | 0.462 |  |
|  | Tumor differentiation (III-IV vs. I-II) | 0.863  (0.567-1.315) | 0.494 | 1.067 (0.736-1.547) | 0.730 |  |
|  | Tumor encapsulation (complete vs. none) | 0.647  (0.462-0.905) | **0.011** | 0.655 (0.479-0.895) | **0.008** |  |
|  | Tumor size, cm (>5 vs. ≤5) | 2.134 (1.518-2.999) | **<0.001** | 2.153 (1.574-2.946) | **<0.001** |  |
|  | Tumor multiplicity (multiple vs. single) | 1.837 (1.235-2.734) | **0.003** | 2.061 (1.410-3.012) | **<0.001** |  |
|  | Vascular invasion (yes vs. no) | 1.393 (0.989-1.961) | 0.058 | 1.620 (1.176-2.230) | **0.003** |  |
|  | TREM2 tumor (positive vs. negative) | 0.525 (0.356-0.773) | **0.001** | 0.553 (0.391-0.781) | **0.001** |  |
| Abbreviations: AFP, [alpha fetoprotein](http://www.iciba.com/alpha_fetoprotein); ALT, alanine aminotransferase; BCLC, Barcelona Clinic Liver Cancer; HR, Hazard ratio; OS, overall survival; TTP, time to progression. | | | | | | |

Supplementary Table 4. Clinical and histopathological features of human tissue specimens enrolled in another different HCC dataset which comprises of 135 HCC cases

| Patients | 135 |
| --- | --- |
| Gender |  |
| Female | 26 |
| Male | 119 |
| Age, years |  |
| ≤52 | 53 |
| >52 | 82 |
| Hepatitis B surface antigen  (positive vs. negative) |  |
| Negative | 23 |
| Positive | 112 |
| ALT (units/L) |  |
| ≤75 | 116 |
| >75 | 19 |
| AFP (ng/mL) |  |
| ≤20 | 49 |
| >20 | 86 |
| Liver cirrhosis |  |
| No | 50 |
| Yes | 85 |
| BCLC stage |  |
| A | 118 |
| B/C | 17 |
| Tumor size (cm) |  |
| ≤5 | 66 |
| >5 | 69 |
| Tumor multiplicity |  |
| Single | 114 |
| Multiple | 21 |
| Tumor encapsulation |  |
| No | 51 |
| Complete | 84 |
| Vascular invasion |  |
| No | 124 |
| Yes | 11 |
| Tumor differentiation |  |
| I-II | 114 |
| III-IV | 21 |

Abbreviations: AFP, [alpha fetoprotein](http://www.iciba.com/alpha_fetoprotein); ALT, alanine aminotransferase; BCLC, Barcelona Clinic Liver Cancer.

Supplementary Table 5. Primers sequences used for qPCR.

| gene | Forward primer | Reverse primer |
| --- | --- | --- |
| TREM2 | GGATGCTGGAGATCTCTGGTT | ATGCAGGCCAGGAGGAGAA |
| PTEN | ACCAGGACCAGAGGAAACCT | GCTAGCCTCTGGATTTGACG |
| E-cadherin | CCCACCACGTACAAGGGTC | ATGCCATCGTTGTTCACTGGA |
| ZO-1 | TGTGAGTCCTTCAGCTGTGGA | GGAACTCAACACACCACCATT |
| CK-18 | GGCATCCAGAACGAGAAGGAG | ATTGTCCACAGTATTTGCGAAGA |
| CK-19 | TGCGGGACAAGATTCTTGGT | TCTCAAACTTGGTTCGGAAGTCA |
| EpCAM | GCAGCTCAGGAAGAATGTG | CAGCCAGCTTTGAGCAAATGAC |
| MMP-2 | TCTTCCCCTTCACTTTCCTG | ACTTGCGGTCATCATCGT |
| MMP-9 | TGGGCTACGTGACCTATGACAT | GCCCAGCCCACCTCCACTCCTC |
| Snail | CACTATGCCGCGCTCTTTC | GGTCGTAGGGCTGCTGGAA |
| Slug | GTCCGTCTGCCGCACCTGAG | ACACGGCGGTCCCTACAGCA |
| Twist | GCCAATCAGCCACTGAAAGG | TGTTCTTATAGTTCCTCTGATTGTTACCA |
| GAPDH | TGCACCACCAACTGCTTAGC | GGCATGGACTGTGGTCATGAG |

Supplementary Table 6. Primer sets for qPCR of *TREM2* gene.

| gene | Forward primer | Reverse primer |
| --- | --- | --- |
| Region 1 (-3000~-4000) | CTTTCCCCAGCAATGGTCAC | ACCTTCCACCTCACATCTGG |
| Region 2 (-3000~-2000) | TGTGACTCTGAGATGGGCAA | GGTGATCTGCCTGGCTCA |
| Region 3 (-2000~-1000) | ACATGGTGATTGTGGTGGTTG | AAGACAGGCCTCCACTCAAA |
| Region 4 (-1000~0) | GAGGGTCCTGGCCTCTAAAG | AGATGGTTGGGGCTGGTAAG |
| Region 5 (0~1000) | CTCTCCGGCTGCTCATCTTA | TGATACTGGGGAGGGAAGGA |
