# Supplementary figures and images for: TREM2 acts as a tumor suppressor in hepatocellular carcinoma by targeting the PI3K/Akt/β-catenin pathway
Source: Oncogenesis. 2019 Jan 25;8(2):9. doi: 10.1038/s41389-018-0115-x (PMC6350080; doi:10.1038/s41389-018-0115-x)

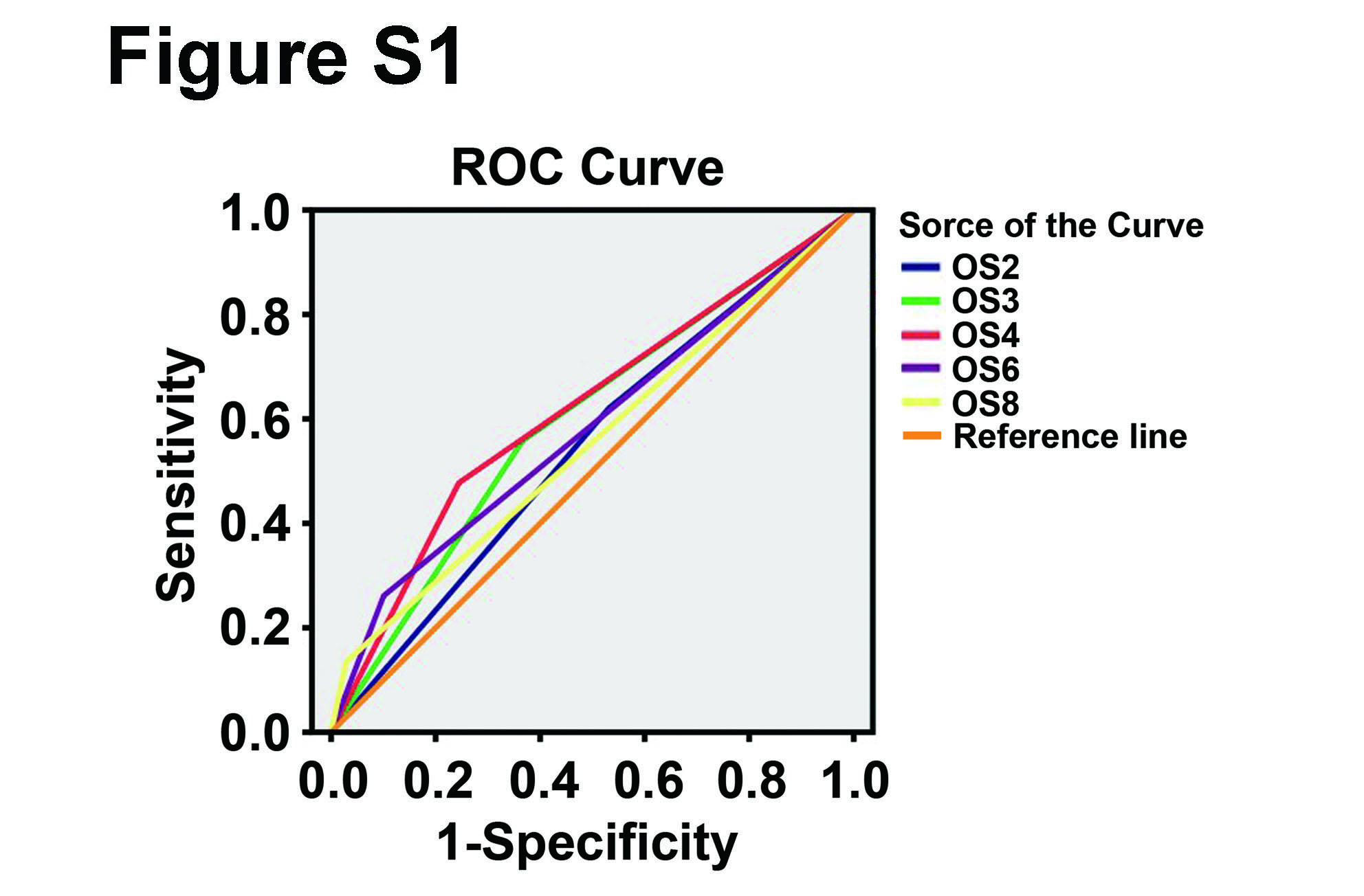

Supplement: Supplementary file 2 — Supplementary figure 1 [file 41389_2018_115_MOESM2_ESM.jpg]

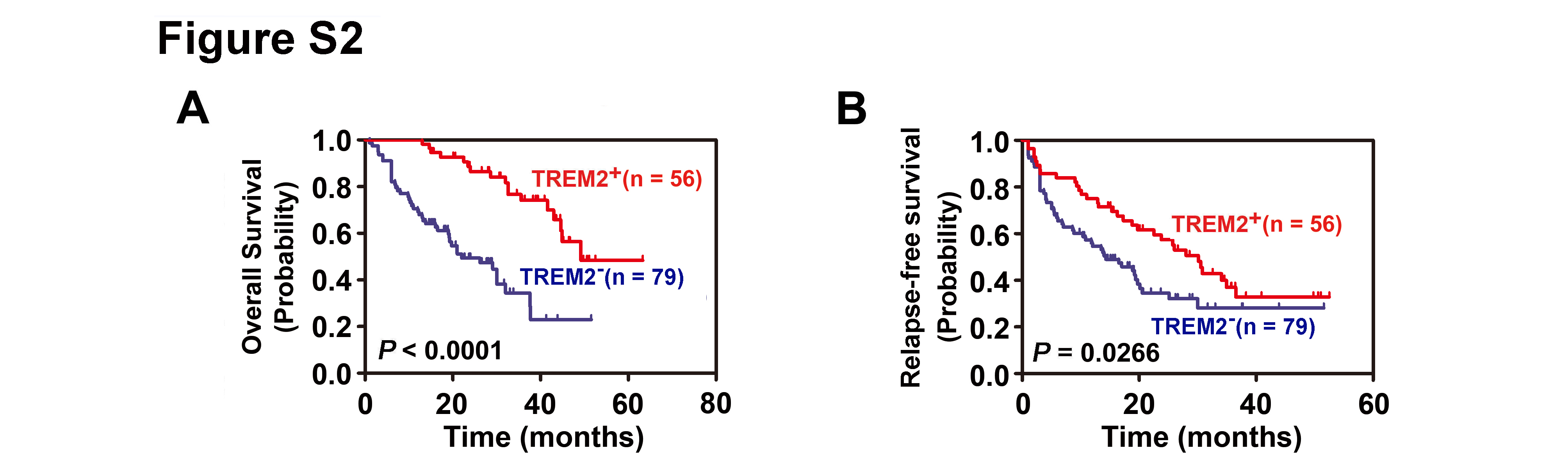

Supplement: Supplementary file 3 — Supplementary figure 2 [file 41389_2018_115_MOESM3_ESM.jpg]

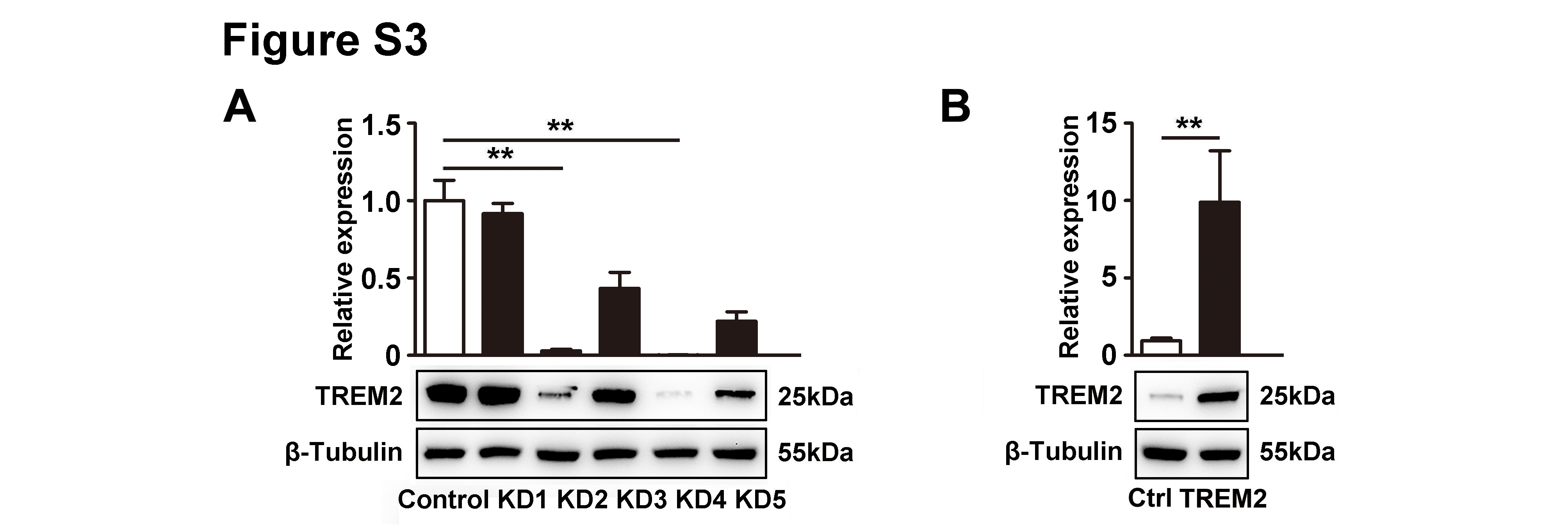

Supplement: Supplementary file 4 — Supplementary figure 3 [file 41389_2018_115_MOESM4_ESM.jpg]

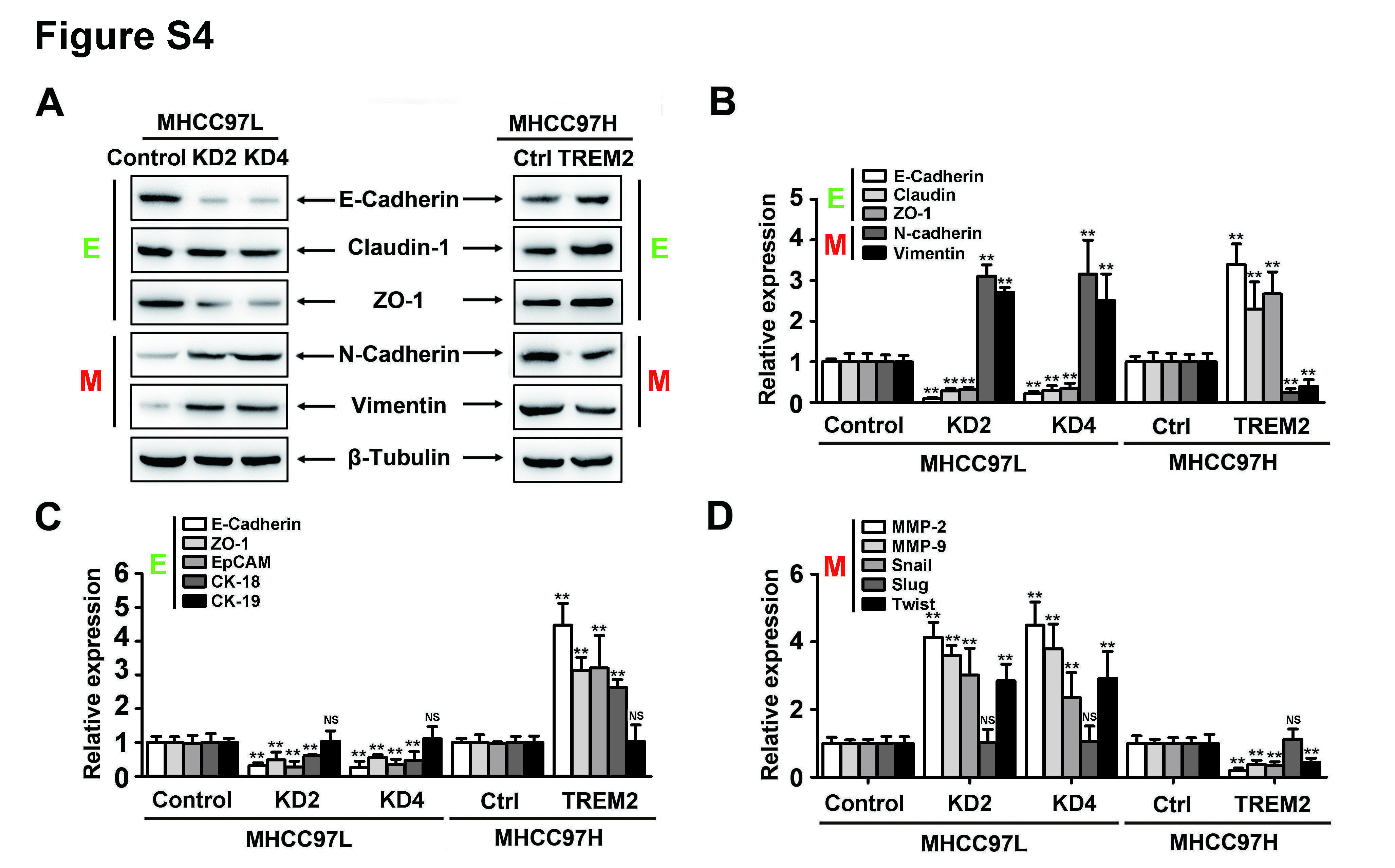

Supplement: Supplementary file 5 — Supplementary figure 4 [file 41389_2018_115_MOESM5_ESM.jpg]

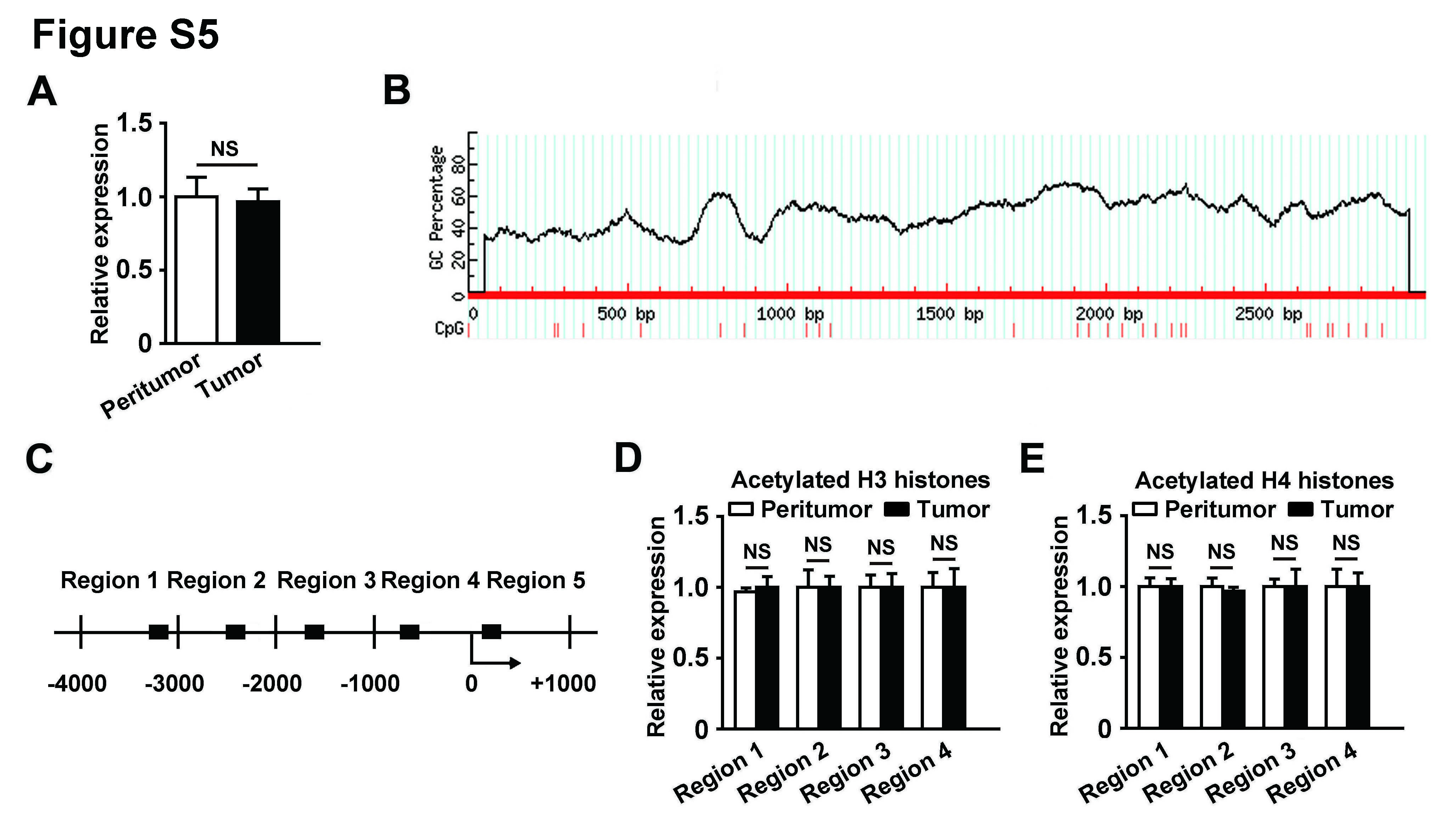

Supplement: Supplementary file 6 — Supplementary figure 5 [file 41389_2018_115_MOESM6_ESM.jpg]

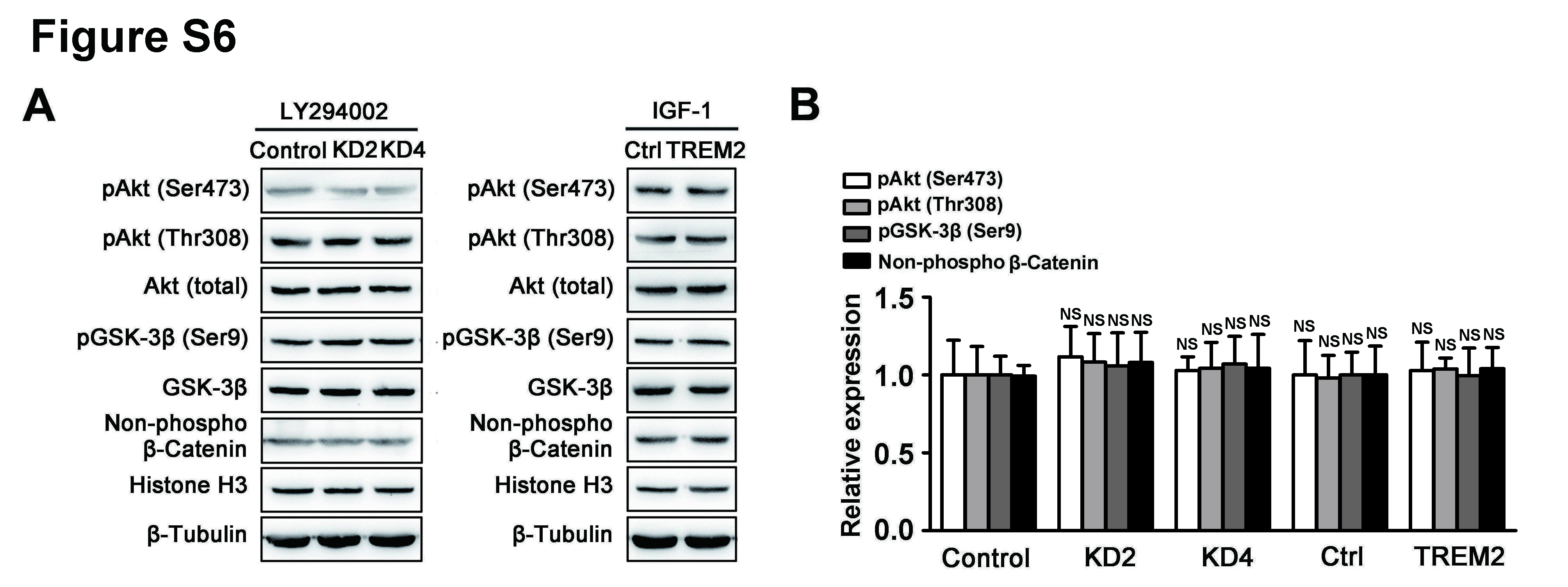

Supplement: Supplementary file 7 — Supplementary figure 6 [file 41389_2018_115_MOESM7_ESM.jpg]

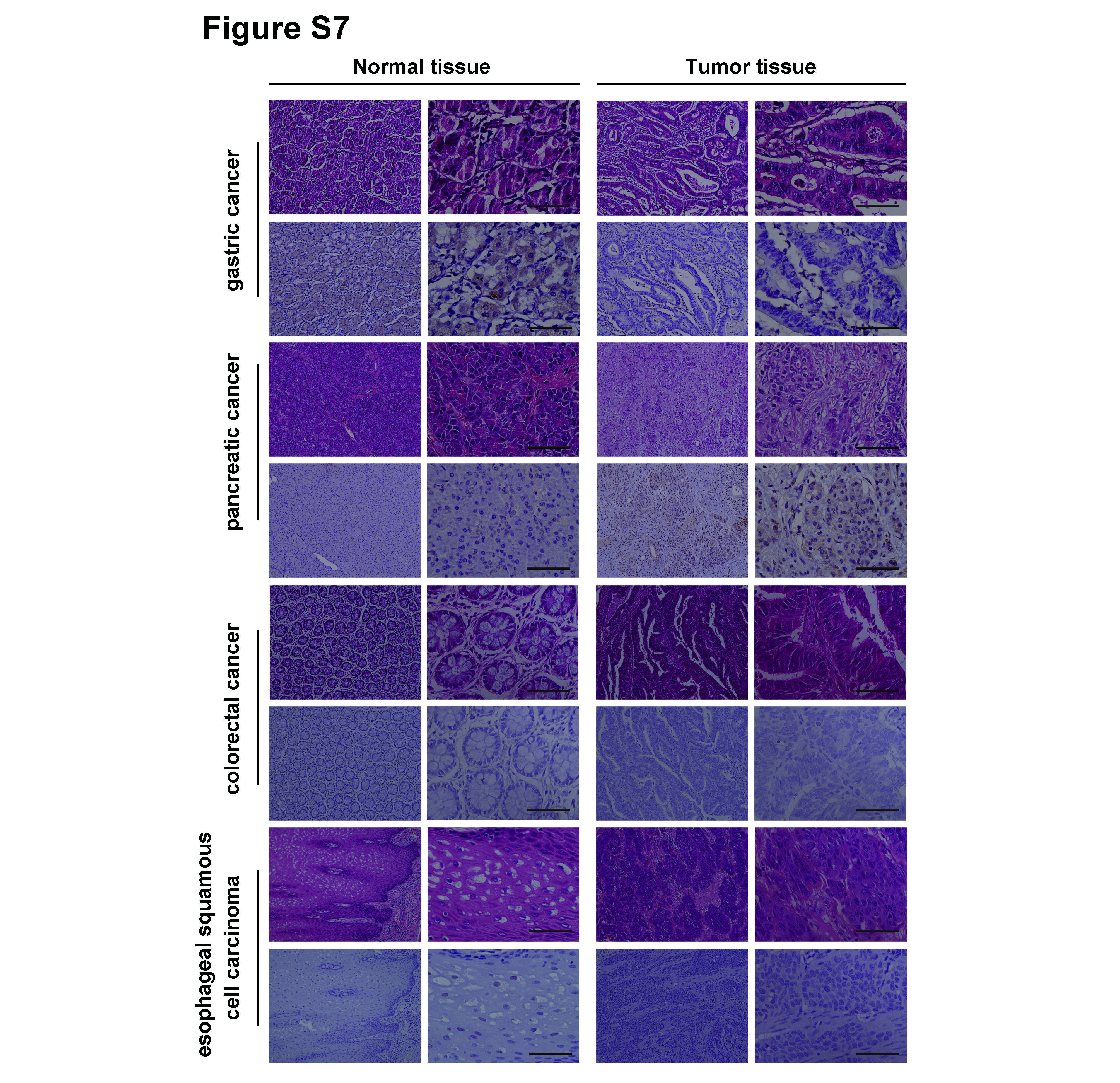

Supplement: Supplementary file 8 — Supplementary figure 7 [file 41389_2018_115_MOESM8_ESM.jpg]
